# Steroid Hormone Signaling Is Essential for Pheromone Production and Oenocyte Survival

**DOI:** 10.1371/journal.pgen.1006126

**Published:** 2016-06-22

**Authors:** Yin Ning Chiang, Kah Junn Tan, Henry Chung, Oksana Lavrynenko, Andrej Shevchenko, Joanne Y. Yew

**Affiliations:** 1 Temasek Life Sciences Laboratory, National University of Singapore, Singapore, Singapore; 2 Howard Hughes Medical Institute and Laboratory of Molecular Biology, University of Wisconsin, Madison, Wisconsin, United States of America; 3 Max Planck Institute of Molecular Cell Biology and Genetics, Dresden, Germany; 4 Pacific Biosciences Research Center, University of Hawai'i at Mānoa, Honolulu, Hawaii, United States of America; Stanford University School of Medicine, UNITED STATES

## Abstract

Many of the lipids found on the cuticles of insects function as pheromones and communicate information about age, sex, and reproductive status. In *Drosophila*, the composition of the information-rich lipid profile is dynamic and changes over the lifetime of an individual. However, the molecular basis of this change is not well understood. To identify genes that control cuticular lipid production in *Drosophila*, we performed a RNA interference screen and used Direct Analysis in Real Time and gas chromatography mass spectrometry to quantify changes in the chemical profiles. Twelve putative genes were identified whereby transcriptional silencing led to significant differences in cuticular lipid production. Amongst them, we characterized a gene which we name *spidey*, and which encodes a putative steroid dehydrogenase that has sex- and age-dependent effects on viability, pheromone production, and oenocyte survival. Transcriptional silencing or overexpression of *spidey* during embryonic development results in pupal lethality and significant changes in levels of the ecdysone metabolite 20-hydroxyecdysonic acid and 20-hydroxyecdysone. In contrast, inhibiting gene expression only during adulthood resulted in a striking loss of oenocyte cells and a concomitant reduction of cuticular hydrocarbons, desiccation resistance, and lifespan. Oenocyte loss and cuticular lipid levels were partially rescued by 20-hydroxyecdysone supplementation. Taken together, these results identify a novel regulator of pheromone synthesis and reveal that ecdysteroid signaling is essential for the maintenance of cuticular lipids and oenocytes throughout adulthood.

## Author Summary

Pheromones are used by many animals to control social behaviors such as mate choice and kin recognition. The pheromone profile of insects is dynamic and can change depending on environmental, physiological, and social conditions. While many genes responsible for the biosynthesis of insect pheromones have been identified, much less is known about how pheromone production is systemically regulated over the lifetime of an animal. In this work, we identify 12 genes in *Drosophila melanogaster* that play a role in pheromone production. We characterized the function of one gene, which we name *spidey*, and which encodes a steroid dehydrogenase. Silencing *spidey* expression during the larval stage results in the rapid inactivation of an essential insect steroid, 20-hydroxyecdysone, and developmental arrest. In adults, *spidey* is needed for maintaining the viability of oenocytes, specialized cells that produce pheromones and also regulate energy homeostasis. Our work reveals a novel role for ecdysteroids in the adult animal and uncovers a regulatory mechanism for oenocyte activity. Potentially, ecdysteroid signaling serves as a mechanism by which environmental or social conditions shape pheromone production. Exploitation of this conserved pathway could be useful for interfering with the mating behavior and lifespan of disease-bearing insects or agricultural pests.

## Introduction

Pheromones, chemical communication signals, profoundly influence the behavior of many organisms [1,2]. For example, the choice to mate or fight in *Drosophila melanogaster* is largely dictated by pheromones [3]. In Lepidoptera, a subtle shift of sex pheromone composition alters mate recognition and has been proposed as one mechanism underlying speciation [4–6]. For many insects, hydrocarbons and other lipids on the cuticle function as pheromones. Circadian rhythm [7], social interactions [8], diet [9,10], and aging [11] are known to alter the cuticular lipid composition. While the biosynthetic pathways for many pheromones are well-defined, only a few molecular modulators have been identified that systemically modulate pheromone production. Namely, the neuropeptide PDF [7], juvenile hormone [12], and insulin-related signals [13] are known to play a role in the regulation of cuticular lipid production.

To discover other modulators of the cuticular lipid profile, we performed a genetic screen using *Drosophila*. In *Drosophila*, the cuticular hydrocarbon (CHC) profiles differ between males and females. The monoene (*Z*)-7-tricosene is the most abundant CHC found on male cuticles and is thought to prevent courtship from other males [14,15]. In contrast, female-specific dienes, (*Z*, *Z*)-7,11-heptacosadeine and (*Z*, *Z*)-7,11-nonacosadiene function as aphrodisiacs for males [14]. The CHCs are synthesized in specialized cells called oenocytes which reside beneath the surfaces of the dorsal and ventral abdomen [16]. Two other male-specific sex pheromones, cis-vaccenyl acetate (cVA) and CH503 (3-O-acetyl-1,3-dihydroxyoctacosa-11,19-diene), are produced in the male ejaculatory bulb [17–19].

Numerous genes encoding enzymes for many of the major CHC biosynthetic steps have been identified including three different desaturases which are needed for carbon-carbon double bond placement [20–23]; elongases [19,24,25] which extend the carbon backbone; several components of the very long-chain fatty acid synthesis pathway which extend the fatty acyl-CoA precursor [26]; fatty acid synthases which produce methyl-branched CHCs [26,27]; and a member of the cytochrome P450 family which catalyzes the final oxidative decarbonylation step common to alkanes and alkenes [28].

To identify novel regulators and components of the pheromone biosynthesis pathway, we carried out a RNA interference (RNAi) screen to knockdown the expression of lipid metabolism genes known to be expressed in the oenocytes and assessed the chemical profile of the cuticles by mass spectrometry (MS). Twelve genes were identified which, upon transcriptional silencing, altered CHC profiles. Further characterization of a predicted steroid dehydrogenase gene *CG1444*, which we name *spidey*, revealed a novel role for ecdysteroid-hormones in the development and survival of adult oenocytes.

## Results

### Mass spectrometry-based screen for modulators of cuticular lipid production

**Fig 1:**
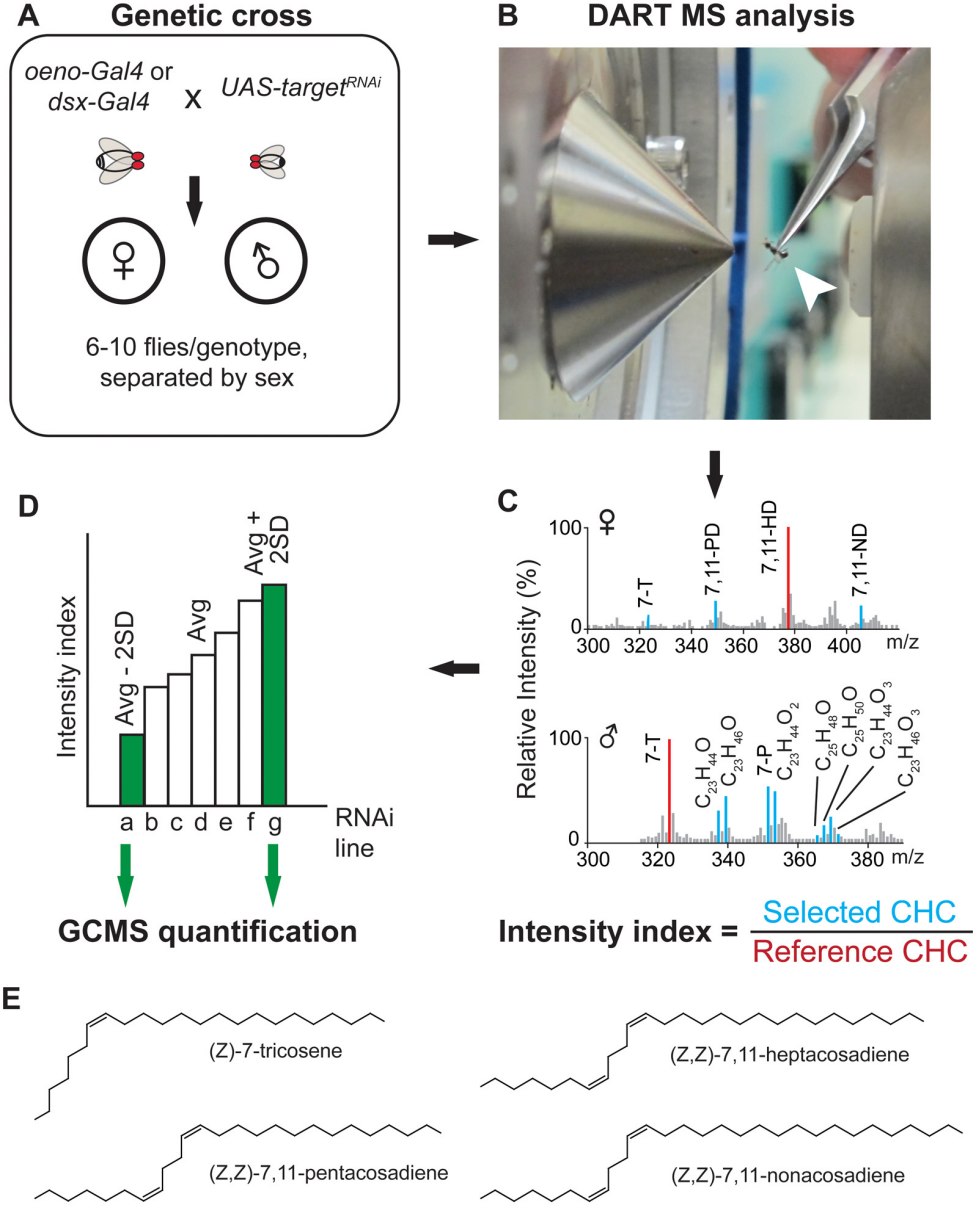
Schematic of RNA interference screen used to identify genes expressed in the oenocytes that contribute to cuticular lipid production. (**A**) Tissue-specific knockdown of the expression of 80 candidate genes was performed using 2 different oenocyte-expressing drivers, *oeno-Gal4* and *dsx-Gal4*. (**B)** Progeny from each cross were assayed individually in the DART MS source (arrowhead). (**C**) Chemical profiles obtained from female and male cuticles. The intensity index for molecules of interest (in blue) is calculated by normalizing signal intensity to an internal reference peak (in red: 7,11-HD for females, 7-T for males); 7-T: (*Z*)-7-tricosene; 7-P: (*Z*)-7-pentacosene; 7,11-PD: (*Z*, *Z*)-7,11-pentacosadiene; 7,11-HD: (*Z*, *Z*)-7,11-heptacosadiene; 7,11-ND: (*Z*, *Z*)-7,11-nonacosadiene. (**D**) Transgenic lines displaying intensity indices which are 2 standard deviations (SD) above or below the average value are subjected to a secondary DART MS screen and quantification by GCMS. (**E**) Chemical structures of the major CHC species characteristic of male or female cuticular profiles.

To identify genes underlying the synthesis of cuticular lipids, we used RNA interference (RNAi) together with the *Drosophila Gal4-UAS* transgene system to silence gene expression within the oenocytes with the *oeno-Gal4* or *dsx-Gal4* driver. *Oeno-Gal4* targets oenocytes while *dsx-Gal4* targets a broad range of tissues including the oenocytes and male ejaculatory bulb (S1 Fig). The relative abundances of individual cuticular lipid species or of each chemical class were compared to the profiles of genetic controls. Eighty genes were selected based on known expression in the oenocytes (from previously published reports) or predicted functionality in lipid metabolism [29–33] (S1 Table). The abundance and composition of major cuticular lipid species were analysed using two forms of mass spectrometry (MS): Direct Analysis in Real Time (DART) MS [34,35] and gas chromatography MS (GCMS). The two MS methods provide complementary detection of, respectively, polar and higher molecular weight molecules and alkanes. Fig 1 illustrates the overall screening strategy.

Following a preliminary round of screening by DART MS and a second confirmatory analysis by GCMS, we identified 12 genes whose functional suppression by RNAi led to quantitative changes in the CHC profiles of females and/ or males (Table 1; S2, S3, S4 and S5 Tables). Three previously known genes underlying hydrocarbon synthesis were identified using our screening strategy: RNAi-mediated gene silencing of *desat1* (*CG5887*) and *desatF* (*CG7923*) produced profiles with significantly lower levels of dienes while knockdown of *eloF* (*CG16905*) produced profiles with overall shorter length CHCs (S2 Fig). The positive identification of known CHC biosynthesis genes validates the sensitivity of the screen design and data analysis.

**Table 1:** Transgenic lines identified by RNAi/ MS screen.

Genotype	Gene name	Proposed function	Modified lipid species^1^
*dsx>CG1444*		Steroid dehydrogenase	♀ 7-T
			♀ 7,11-PD
*dsx>CG2781*		Fatty acid elongase	♀ 7-T
			♂ Me-branched
			♂ Oxy-CHCs
*dsx>CG6300*		Long-chain fatty acid transporter	♀ 7,11-ND
			♀ Me-branched
*dsx>CG1765*	*EcR*	Ecdysone receptor	♂ Monoenes
			♂ Me-branched
			♂ Oxy-CHCs
*dsx>CG7400*	*Fatp*	Long-chain fatty acid transporter	♂ Monoenes
*dsx>CG11140*	*Aldh-III*	Aldehyde dehydrogenase type III	♂ Monoenes
*oeno>CG7400*	*Fatp*	Long-chain fatty acid transporter	♀ 7,11-ND
			♂ Monenes
*oeno>CG1444*		Steroid dehydrogenase	♂ Monoenes
*oeno>CG2781*		Fatty acid elongase	♂ Me-branched
			♂ Oxy-CHCs
*oeno>CG3961*		Long-chain fatty acid-coA ligase	♂ Oxy-CHCs
*oeno>CG5162*		Carboxylic ester hydrolase	♂ alkanes
			♂ Oxy-CHCs
*oeno>CG8522*	*SREBP*	Sterol regulatory element binding protein	♂ Monoenes
*oeno>CG9102*	*bab2*	Transcription factor	♀ 7,11-ND
			♀ Me-branched
*oeno>CG11502*	*seven up*	Steroid hormone receptor	♀ 7,11-PD
*oeno>CG17562*		Fatty-acyl-CoA reductase	♀ 7,11-ND
			♀ Me-branched

^1^Oxy-CHCs: oxygenated CHC species; 7-T: (*Z*)-7-tricosene; 7, 11-ND: (*Z*, *Z*)-7, 11-nonacosadiene; 7, 11-PD: (*Z*, *Z*)-7, 11-pentacosadiene; monoenes include tricosene, pentacosene, and heptacosene; Me-branched: methyl branched alkanes include 2-MeC24, 2-MeC26, 2-MeC28, and 2-MeC30. All measurements were verified with DART MS and GCMS measurements.

For several transgenic lines, gene silencing resulted in a shift towards longer CHCs in both males and females (*e*.*g*., *CG7400*) or overall shortening (*CG11140*, in males). In other lines, select chemical classes were altered. For example, the abundance of alkanes increased upon knockdown of *CG5162* while silencing of *CG2781*, *CG1765*, or *CG1444* resulted in higher levels of methyl branched alkanes in males. In females, knockdown of *CG9102*, *CG6300*, or *CG17562* led to lower levels of monoenes and a relative increase of long chain alkanes and nonacosadiene. In males, knockdown of *CG5162*, *CG3961*, *CG2781*, or *CG1765* resulted in decreased levels of oxygen-containing CHCs. Developmental lethality was observed with two lines: *CG6660* (consistent with its recently reported role in tracheal waterproofing [36]), and *CG1444*.

### *Spidey* misexpression during development causes pupal lethality

**Fig 2:**
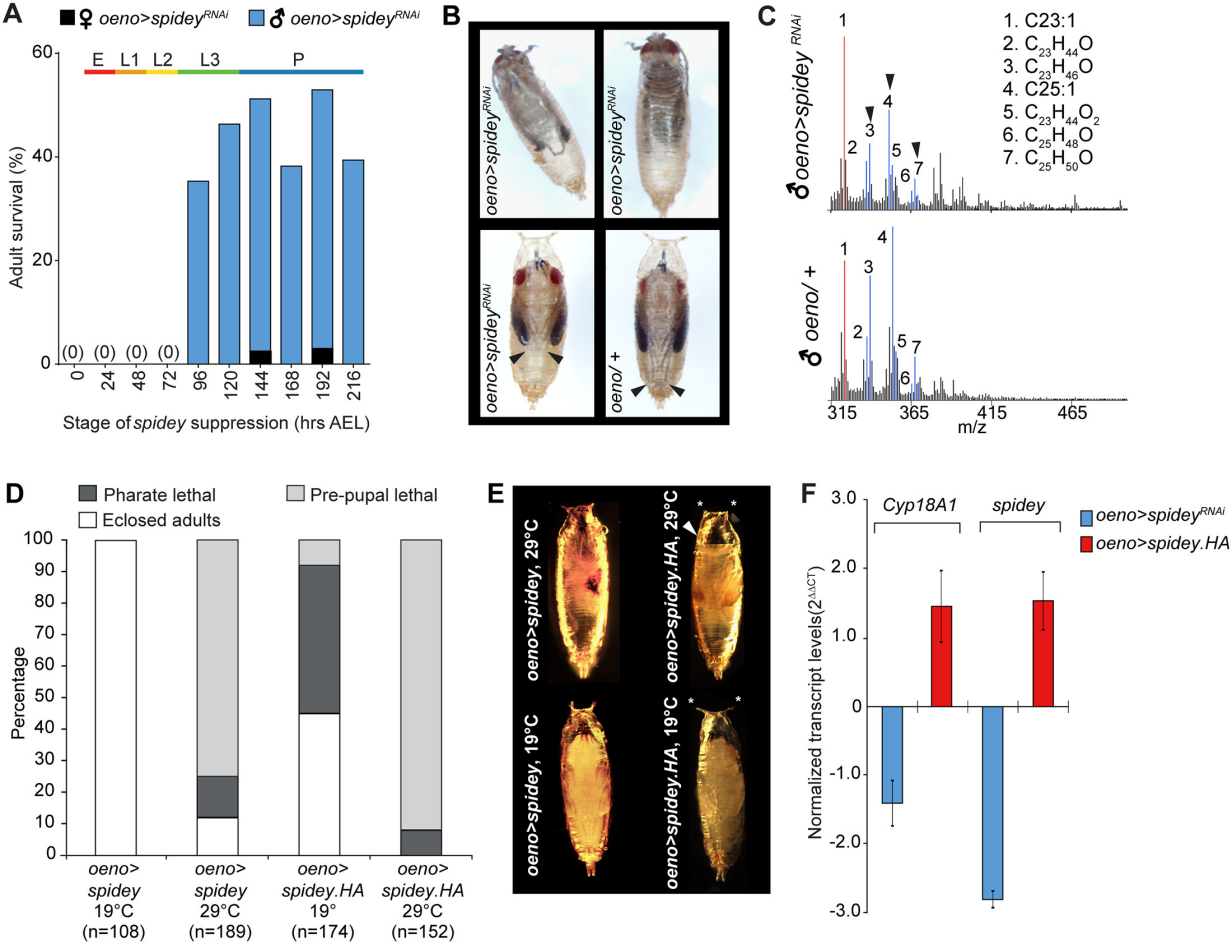
*Spidey* expression during development is essential for adult survival. (**A**) Silencing *spidey* expression from 0–72 hours after egg laying (AEL) results in pharate lethality of males and females. Male survivorship significantly improves when *spidey* is suppressed from 96 hours AEL but few females successfully eclose. Survival % is calculated as the number of successfully eclosed adults relative to the total number of male and female embryos; E: embryo; L1: 1^st^ instar larvae; L2: 2^nd^ instar larvae; L3: 3^rd^ instar larvae; P: pupae. N = 50 embryos for each stage. See S6 Table for full genotypes. (**B**) Top: *oeno>spidey*^*RNAi*^ flies fail to emerge from the pupal case. Bottom left: malformed legs (arrowheads) are shorter in *oeno>spidey*^*RNAi*^ males and females compared to genetic controls (bottom right). (**C**) Representative DART MS spectra from *oeno>spidey*^*RNAi*^ males reveals an overall decrease of major cuticular lipid signals (blue, arrows) relative to tricosene (peak 1, red). In spectra from control lines, pentacosene (peak 4) and tricosene (peak 1) exhibit similar relative intensity levels. (**D**) Continuous overexpression of *spidey* at 29°C during development results in significant pre-pupal and pharate lethality compared to controls raised at 19°C (p<0.0001 for *oeno>spidey* and *oeno>spidey*.*HA*, compared to controls, Chi-square test, n = 189). (**E**) Top: *oeno>spidey* and *oeno>spidey*.*HA* overexpression lines exhibit pre-pupal lethality and defects in head (arrowheads) and spiracle (*) eversion. Bottom: controls raised at 19°C. (**F**) Knockdown or overexpression of *spidey* at 29°C alters *Cyp18a1* and *spidey* transcript levels relative to control levels measured at 19°C. *Rp49* was used as an internal control for normalization. Data represent the average of 3 experimental replicates ± SEM.

Because of its dual role in metamorphosis and adult cuticular lipid production, we further investigated the function of *CG1444*, which we name *spidey*. Based on amino acid sequence similarity, *spidey* is predicted to function as a steroid dehydrogenase, a well-conserved enzyme class that plays a prominent role in converting inactive keto steroids to hydroxy steroids in a diversity of organisms [37,38]. To characterize the role of *spidey* during development, we silenced its expression in the oenocytes of transgenic flies (*oeno>spidey*^*RNAi*^) at various time points after embryo fertilization with the use of *tubulin-Gal80*^*ts*^, a ubiquitously-expressed temperature-sensitive transgene. The Gal80^ts^ protein suppresses Gal4 function at 19°C and permits binding to the UAS element at 29°C. Embryos were shifted to the restrictive temperature at successive 24 hour intervals after egg laying (AEL). Knockdown of *spidey* in oenocytes before the mid-3^rd^ instar stage caused complete pharate lethality of both male and female flies (Fig 2A and 2B). In contrast, *spidey* knockdown after the mid-3^rd^ instar resulted in successful eclosion of most males and only very few females (Fig 2A). Females failed to eclose during the late pupal stage and 80% of the pupae exhibited a malformed leg phenotype (Fig 2B; p<0.0001, compared to controls; Fisher Exact Probability test, N = 36), a feature that is also found in flies with defects in ecdysone metabolism [39]. In contrast, male survivors exhibited a 1–2 day delay to pupal formation and a significant overall decrease of many of the major cuticular lipids relative to genetic controls (Fig 2C).

Overexpression of *spidey* in the oenocytes also resulted in developmental lethality. At 29°C, misexpression with 2 different *UAS* constructs (*oeno>spidey* and *oeno>spidey*.*HA*) both resulted in 87% early pupal or pharate lethality (Fig 2D and 2E). At the permissive temperature, all *spidey* flies and 45% of *spidey*.*HA* pupae successfully eclosed. Quantitative PCR of *spidey* transcript levels in *oeno>spidey*^*RNAi*^ and *spidey*.*HA* flies confirmed, respectively, a decrease and increase in expression levels at the restrictive temperature (Fig 2F).

The phenotypes observed following misexpression or knockdown of *spidey* were reminiscent of ecdysone pathway mutants. In particular, the malformed legs, partial eclosion, and defective head and spiracle eversion phenotypes have been observed upon overexpression or loss-of-function of the ecdysone inactivating enzyme Cyp18a1 [39]. To determine if manipulation of *spidey* affects *Cyp18a1* expression, we quantified *Cyp18a1* transcript levels following *spidey* knockdown or overexpression. *Oeno>spidey*^*RNAi*^ larvae exhibited a 1.4-fold decrease in *Cyp18a1* expression whereas *oeno>spidey*.*HA* larvae showed a 1.5 fold increase (Fig 2F). Taken together, these results indicate that *spidey* expression influences expression of *Cyp18a1*, a key component of the ecdysone metabolic pathway.

### *Spidey* misexpression disrupts steroid hormone metabolism

Since the product of *spidey* is predicted to have steroid dehydrogenase activity and both its knockdown and overexpression result in phenotypic features characteristic of ecdysone signaling disruption, we hypothesized that *spidey* misexpression alters ecdysteroid titres, resulting in pharate lethality. We examined the steroidal profile of flies in which *spidey* is silenced with the GeneSwitch system [40]. Control and experimental lines have the same genetic background (*GSoeno>spidey*^*RNAi*^) but RNAi expression in the oenocytes (and hence, knockdown of *spidey*) is activated only by exposure to the exogenously applied ligand RU-486. Hormonal extracts were compared between larvae fed RU-486 (*spidey*^*KD*^) and control animals that were not fed the drug (*spidey*^*control*^). By comparing flies with the identical genotype, variation in steroid levels due to genetic background is avoided.

**Fig 3:**
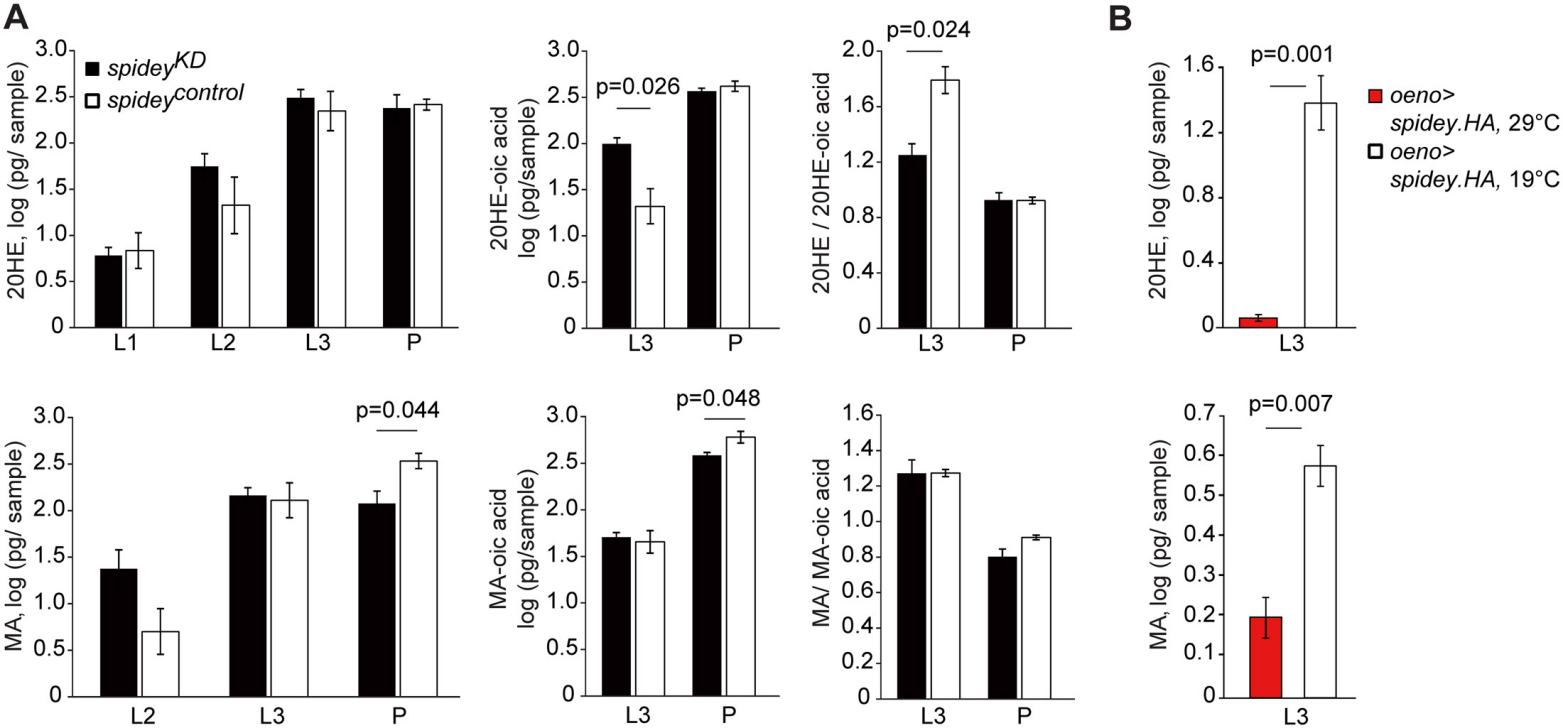
Quantification of ecdysteroids and ecdysonic acids metabolites by LC-MS/MS. (**A**) Top row: 20HE-oic acid levels were significantly elevated in *spidey*^*KD*^ L3 larvae, resulting in a lower 20HE to 20HE-oic acid ratio. 20HE-oic acid was not detected in L1 and L2 animals. Bottom row: makisterone A (MA) and makisteronoic acid (MA-oic acid) levels were lower in *spidey*^*KD*^ animals compared to *spidey*^*control*^ at mid-pupal stage (P). However, the ratio of MA to MA-oic acid in pupal stage was not significantly different. (**B**) Following overexpression of *spidey*, L3 larvae exhibit a significant decrease of 20HE and MA compared to controls. Data represent the average log-transformed values of 3 experimental replicates ± SEM; Student’s t-test.

The analysis of extracts from larval and pupal development stages by liquid chromatography MS/MS (LC-MS/MS) showed that the steroid hormones 20-hydroxyecdysone (20HE), makisterone A (MA), and their respective catabolic products, 20HE-oic acid and MA-oic acid [41,42], were altered in *spidey*^*KD*^ flies compared to controls (Fig 3A). While overall levels of 20HE were not changed, both 20HE-oic acid and the ratio of 20HE to 20HE-oic acid were significantly altered in *spidey*^*KD*^ 3^rd^ instar larvae, indicating that reduced *spidey* expression results in the accelerated oxidation of 20HE. A small effect on MA and its catabolic product was also observed in the pupal stage though no significant difference was found in the MA/ MA-oic ratio. In contrast, overexpression of *spidey* resulted in a substantial drop in 20HE and MA levels in 3^rd^ instar larvae (Fig 3B). Levels of the metabolites 20HE-oic acid and MA-oic acids were below the limits of detection. To test whether *spidey-*induced changes in ecdysteroid levels could underlie the developmental defects, we supplemented fly food with 20HE, cholesterol, or fatty acids. However, none of these manipulations were able to rescue the larval lethality phenotype of *oeno*>*spidey*^*RNAi*^ flies (S3 Fig).

### *Spidey* is essential for CHC synthesis in adult flies

**Fig 4:**
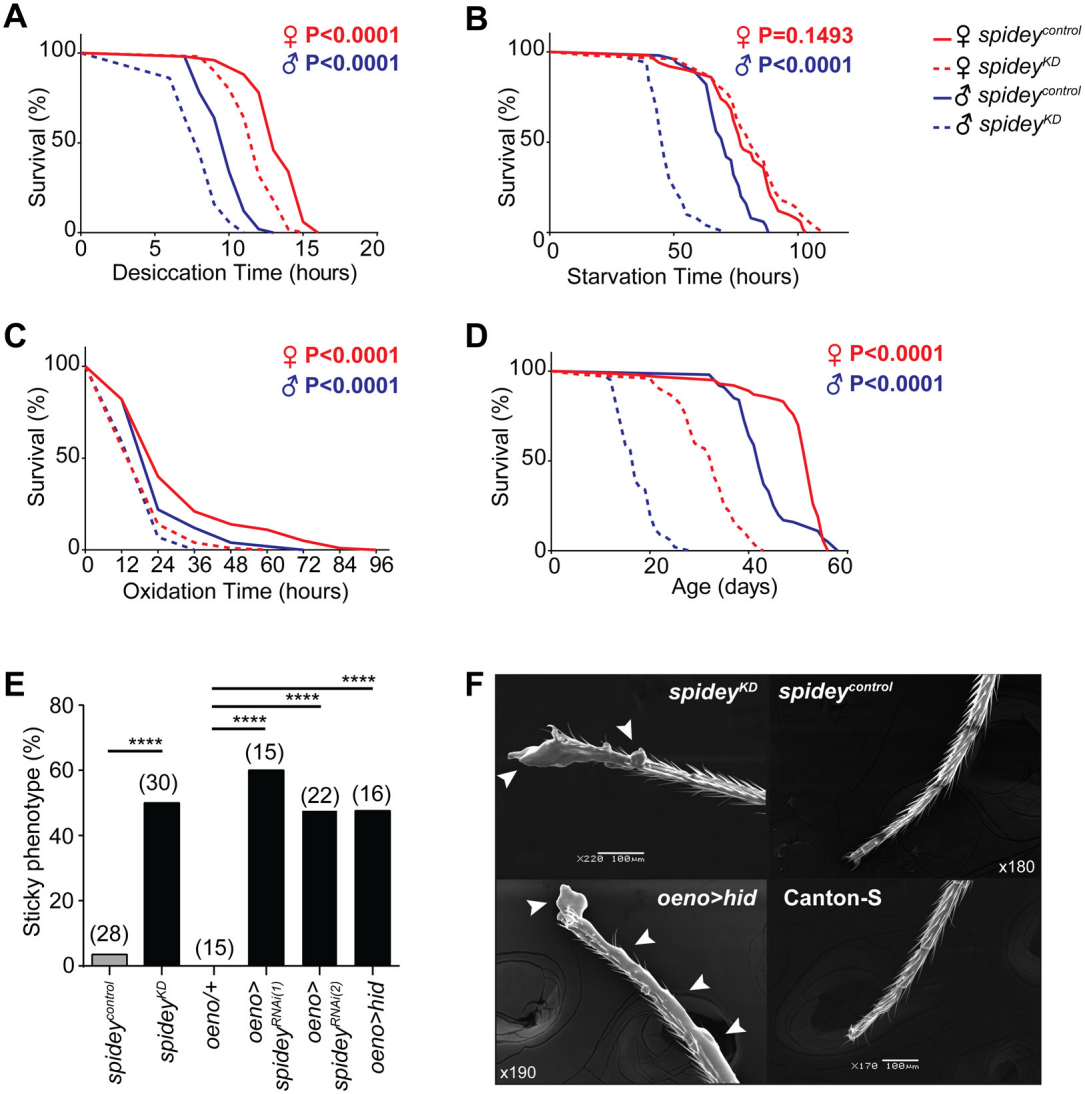
*Spidey* suppression leads to compromised fitness, longevity, and “Spider-Man” phenotype. (**A**) *Spidey*^*KD*^ males and females (dashed lines) exhibit poorer desiccation resistance compared to genetically identical *spidey*^*control*^ flies (solid lines); N = 50 flies for all treatments, P-values comparing experimental to control flies were obtained by log-rank test. See S6 Table for full genotypes. (**B**) *Spidey*^*KD*^ males are less resistant to starvation compared to genetically identical control flies. N = 50 flies for all treatments, P-values comparing experimental to control flies were obtained by log-rank test. (**C**) *Spidey*^*KD*^ males and females exhibit poorer oxidation resistance compared to genetically identical control flies; N = 50 flies for all treatments, P-values comparing experimental to control flies were obtained by log-rank test. (**D**) *Spidey*^*KD*^ males and females exhibit a shorter lifespan compared to genetically identical control flies; N = 100 flies for all treatments, P-values comparing experimental to control flies were obtained by log-rank test. (**E**) Flies exhibiting the sticky “Spider-man” phenotype are found dead on the vertical wall of the vial and do not detach despite vigorous shaking and knocking. The sticky phenotype was observed in 50–60% of males with suppressed *spidey* expression (*spidey*^*KD*^, 14–19 days old; *oeno>spidey*^*RNAi*^, 20–27 days old) or genetically ablated oenocytes (*oeno>hid*, 13–17 days old). Only 1 fly from the age-matched control groups (*spidey*^*control*^, 47–56 days old; *oeno/+*, 53–62 days old) exhibited the sticky phenotype; one tailed Fisher’s exact test, ****p<0.0001; sample size is shown above each bar. (**F**) Representative electron micrographs showing the accumulation of food residue (indicated by arrowheads) on the legs of flies exhibiting the sticky phenotype (*spidey*^*KD*^, *oeno>hid)*. Control (*spidey*^*control*^) and wildtype Canton-S flies do not appear to have residue on the legs.

Ecdysteroids in adults are known to play a significant role in organ viability, aging, and stress resistance [43]. We next asked whether the loss of *spidey* function during adulthood also affects various life history traits. Gene expression was manipulated in *spidey*^*KD*^ adults by feeding RU-486 at various time points after eclosion. Interestingly, *spidey*^*KD*^ flies showed a decreased resistance to desiccation, lowered starvation resistance, increased susceptibility to oxidative stress, and shortened lifespan (Fig 4A–4D).

We noted that approximately 50% of male *spidey*^*KD*^ flies exhibited an unusual phenotype at from 14–19 days old. Flies were found attached to the walls of the food vials and could not be displaced by vigorous shaking and knocking (S1 Movie). Flies appeared to die from starvation as a result of not being able to remove themselves from the walls. The “sticky” phenotype was also observed using a second RNAi line (11 out of 22 flies, age 25 days; Fig 4E). Scanning electron micrographs identified a layer of food-like substance coating the tarsal segments of *spidey*^*KD*^ fly legs but not on *spidey*^*control*^ or wildtype fly legs (Fig 4F). Because of the striking ability of *spidey*^*KD*^ flies to adhere to vertical surfaces, we named this gene “*spidey*”, as an homage to the comic book character, Spider-Man.

**Fig 5:**
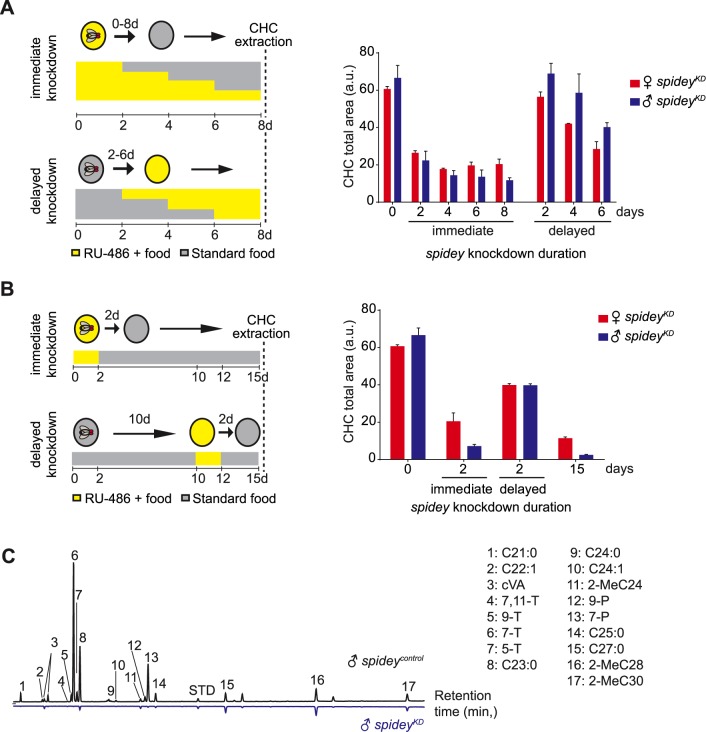
*Spidey* is essential for cuticular lipid synthesis in adult flies. (**A**) Two different feeding regimes of adult flies are used to produce an immediate or delayed knockdown of *spidey* expression. Gene suppression is achieved by feeding RU-486 to *GSoeno>spidey*^*RNAi*^ flies; suppression is removed by placing flies on standard food. Cuticular lipids are extracted at 8 days old. A 65–85% decrease in CHC levels is apparent when *spidey* is suppressed using the immediate knockdown regime but not the delayed regime. Data represent the mean intensity of 3 experimental replicates (8 flies each) ± SEM; Student’s t-test, *p<0.05; a.u.: arbitrary units. See S6 Table for full genotypes. (**B**) Immediate and delayed knockdown of *spidey* over 15 days. The CHC levels do not recover at 15 days when *spidey* is suppressed from age 0–2 days. *Spidey* suppression between age 10–12 days produces an intermediate effect on CHC levels. Data shown represent the mean ± SEM from 3 experimental replicates with 8 flies each); Student’s t-test, *p<0.05. (**C**) Representative GCMS chromatograms of cuticular lipid extract from 15 day old *spidey*^*control*^ and *spidey*^*KD*^ males. Major peaks are labelled with the number of carbon atoms followed by number of double bonds; T: tricosene; P: pentacosene; STD: C26:0 standard spiked into the hexane solvent.

We hypothesized that the loss of hydrophobicity and increased susceptibility to environmental stressors could be due to a defect of the waxy cuticular surface. Quantitative analysis of CHC amounts by GCMS revealed that first, CHC levels decreased as the duration of *spidey* knockdown increased; and second, males were more severely affected. At 8 days old, *spidey*^*KD*^ adults continuously exposed to RU-486 showed a reduction in overall CHCs levels with an 85% decrease for males and 65% decrease for females compared to age-matched *spidey*^*control*^ flies (Fig 5A). Notably, the most dramatic effects were observed when *spidey* knockdown occurred immediately after eclosion: s*pidey* suppression only in the first 0–48 hrs post-eclosion produced as large an effect on CHC loss as continuous suppression of the gene for 8 days (Fig 5A). Moreover, the CHC levels in early adulthood knockdown animals were not restored at 15 days old even though the flies were returned to standard food conditions (hence, alleviating inhibition of *spidey*) from days 3–15 (Fig 5B). Knockdown of the gene for 48 hrs only at 10 days of age produced an intermediate decrease in CHC levels while prolonged suppression for 15 days led to near complete loss of all detectable CHCs (Fig 5B and 5C). These results indicate that *spidey* is needed throughout adult life for the maintenance of CHCs but is critical in early adult life to establish normal CHC levels. Consistent with this observation, *spidey* gene expression is maintained at a constant level throughout adulthood (S4 Fig).

**Fig 6:**
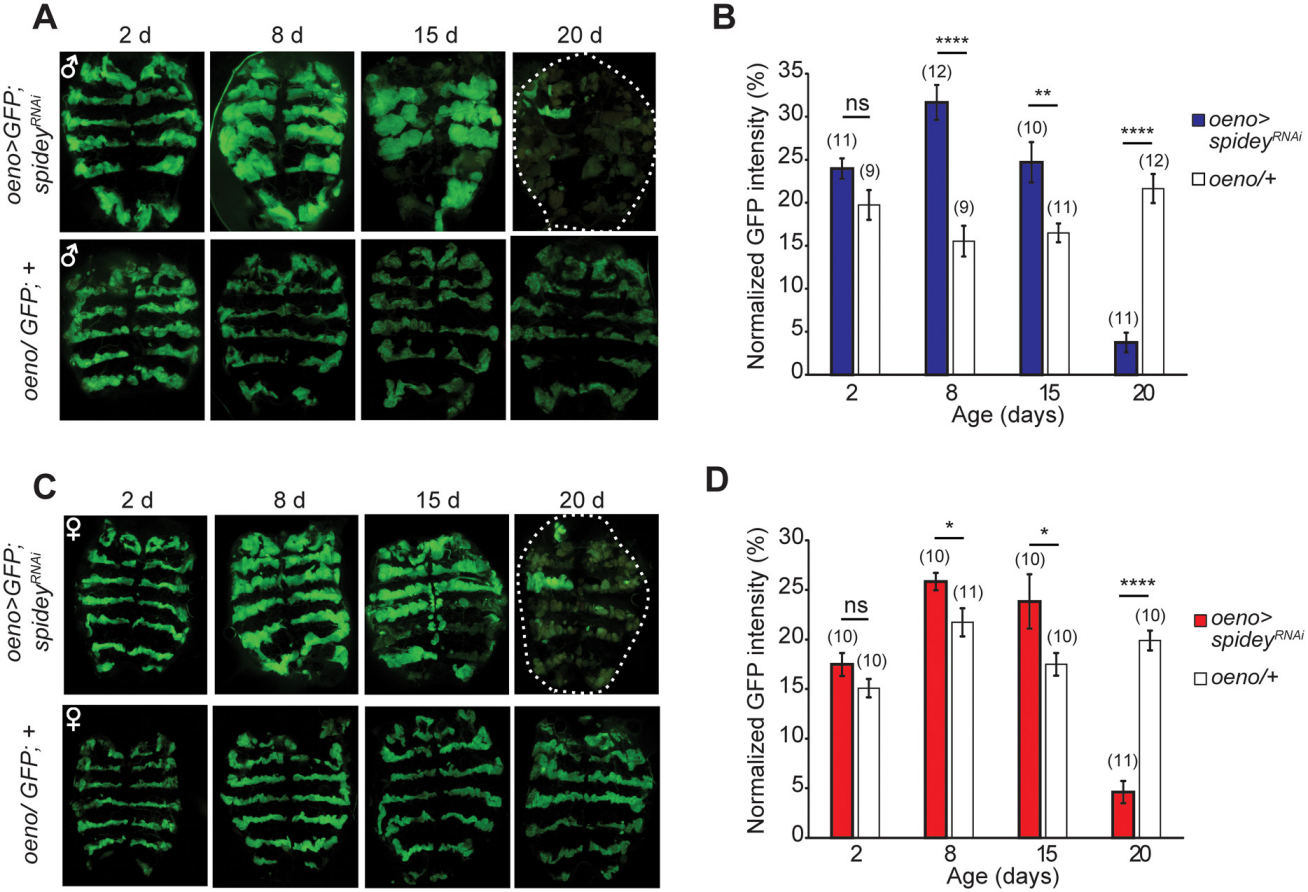
*Spidey* is necessary for oenocyte viability in aged adults. (**A**) Fluorescent microscope images of GFP-labeled oenocytes in males reveal that cellular morphology and cell numbers change upon silencing of *spidey* in adult flies. At 8 and 15 days old, oenocytes appear swollen in *spidey*-suppressed flies compared to controls. At 20 days old, very few GFP-positive cells were observed. See S6 Table for full genotypes. (**B**) Quantification of GFP intensity in oenocytes reveals a significant increase at 8 and 15 days old in *spidey-*suppressed flies followed by a dramatic decrease at 20 days old. Sample sizes are indicated above each bar; one-way ANOVA with Tukey’s multiple comparisons test; **: p<0.01, ****: p<0.0001; ns: not significant. (**C**) Fluorescent microscope images of GFP-labeled oenocytes in *spidey*^*KD*^ females reveal similar morphological changes as observed with males. (**D**) Quantification of GFP intensity in oenocytes reveals a slight but significant increase at 8 and 15 days old in *spidey* suppressed females followed by a dramatic loss at 20 days old; samples sizes are indicated above each bar. One-way ANOVA with Tukey’s multiple comparisons test; *: p<0.05, ****: p<0.0001; ns: not significant.

The loss of CHCs and ensuing sticky phenotype could be due to a defect in biosynthesis or a loss of CHC-producing oenocytes. However, total fatty acid amounts were similar or even higher in both *spidey*^*KD*^ males and females compared to controls (S5 Fig), indicating that a reduction in fatty acid precursors is not likely to be the main cause of CHC loss. Ubiquitous knockdown of *spidey* using a *GStub-Gal4* driver also showed no difference in total fatty acid levels or individual fatty acid species compared to genetic controls (S5 Fig). To directly investigate oenocyte viability, we visualized the morphology and quantified the number of adult oenocytes by expressing a membrane-bound green fluorescent protein (GFP). At 8 days old, GFP intensity in *spidey-*suppressed flies increased significantly due to a swollen appearance of the membrane (Fig 6). By 15 days old, both female and male flies exhibited a mix of swollen cells, large patches devoid of oenocyte GFP signal, and an overall decrease of GFP expression on the dorsal and ventral abdomen. By 20 days old, large swaths of oenocytes appeared to be missing. Taken together, these data indicate that *spidey* is required to maintain the viability of oenocytes in adult flies. Silencing *spidey* expression reduces oenocyte numbers and produces a severe loss of CHC in aged *spidey*^*KD*^ flies with concomitant deficits in stress resistance, lifespan, and hydrophobicity. Consistent with this observation, ablation of oenocytes by expression of the pro-apoptotic gene *hid* during late pupal stage also resulted in the *spidey* wall-sticking phenotype (Fig 4E).

**Fig 7:**
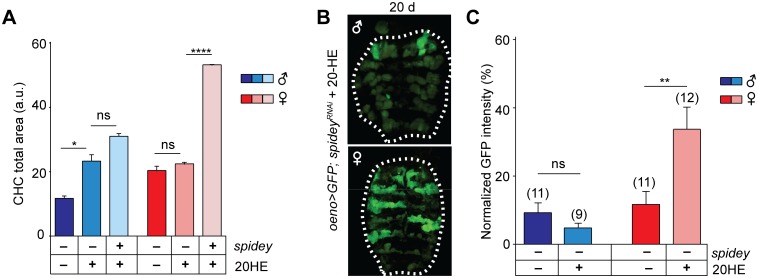
Partial rescue of *spidey* defects by 20HE. (**A**) At 8 days old, 20HE supplementation restores total CHC levels to near control conditions in male but not female *spidey*^*KD*^ flies. Data shown are the mean ± SEM from 3 experimental replicates with 8 flies each; one-way ANOVA with Tukey’s multiple comparison test, *p<0.05, ns: not significant; a.u.: arbitrary units. See S6 Table for full genotypes. (**B**) At 20 days old, GFP signal intensity of oenocytes in *oeno>spidey*^*RNAi*^ males remains low and aberrantly distributed despite 20HE supplementation. (**C**) Females exhibit a significant increase in GFP intensity with 20HE feeding. Mean GFP intensity ± SEM shown; samples sizes are indicated above; Student’s t-test, **: p<0.01; ns: not significant.

To determine whether the decreased availability of 20HE due to accelerated oxidation underlies the reduction of CHC levels in *spidey*^*KD*^ adults, we attempted to rescue the deficits by adding 20HE to the adult diet of *spidey*^*KD*^ flies. Interestingly, the CHC levels of *spidey*^*KD*^ males but not females were restored to near-control levels upon steroid supplementation at 8 days old (Fig 7). However, at 20 days old, males still exhibited massive loss of oenocytes indicating that the rescue effects of 20HE are only temporary. While 20HE did not rescue CHC levels in female *spidey*^*KD*^ flies, ecdysteroid supplementation appeared to improve oenocyte viability at 20 days old, based on GFP intensity (Fig 7B and 7C). Taken together, the findings show that 20HE is able to partially rescue some aspects of oenocyte function however other signals are likely needed for sustained viability and function.

## Discussion

Using a screening approach that combines RNAi and mass spectrometry, we identified twelve genes that contribute to cuticular lipid synthesis. Two methods of mass spectrometry were used, each allowing analysis of overlapping but non-identical chemical classes of small molecules. DART MS is capable of detected oxygenated CHCs, a lipid species that is not observed under standard GCMS conditions. In contrast, GCMS but not DART MS is able to detect alkanes and differentiate between isobaric compounds. Alkenes are readily detected by both methods. We used DART MS as a preliminary screening tool since fewer flies and minimal sample preparation are required to obtain a rapid, semi-quantitative measure of cuticular lipids. However, due to the analytical limitations of DART ionization, genes involved in alkane and branched-alkane production are likely to have been missed. Nevertheless, the GCMS measurements largely agreed with data from DART MS. Importantly, the parameters of the screen proved to be sufficiently sensitive for identifying transgenic lines that exhibit abnormal cuticular lipid profiles.

### Pheromone profiles reflect changes in lipid metabolism and steroid signaling

Pheromone production in insects utilizes many of the same biosynthetic routes and cellular sites integral to lipogenesis [44]. In *Drosophila*, pheromone-producing oenocytes play essential roles in fat storage and release, similar to some of the roles of mammalian hepatocytes [33]. As such, the chemical composition of the cuticle can reflect changes in lipid metabolism due to aging [11,13], reproductive state [45], diet [9,10,46,47], and environmental conditions [48,49]. In addition to identifying components of the fatty acid synthesis pathway, our screen also found genes with broad functionality including a master regulator of lipid synthesis (*SREPB*) [50] and the transcription regulatory factor *bric à brac (bab2)* [51]. These candidate genes could serve as mechanistic links by which environmental and physiological changes modulate lipid pheromone metabolism. In particular, genes that were found to influence levels of oxygenated CHCs will be of interest for future studies since the abundance of these molecules changes in response to age and insulin-related signaling [13].

Our screen also identified three sterol-related genes. Previously, juvenile hormone was shown to be involved in CHC regulation [12,52–54]. Our results found that ecdysteroid hormones are important not only for maintaining levels of CHCs in adults but are needed, as well, for the viability of oenocytes. Even a brief abatement of *spidey* activity during early adulthood was sufficient to cause lasting changes in the cuticular lipid profile late into adulthood. Ecdysteroids have been shown to alter phenotypic and life history traits such as pigmentation, life span, stress resistance [55], and reproduction [56] in response to environmental cues. Our results indicate that the pheromone profile is another trait subject to steroid control.

### Role of *spidey* in ecdysteroid metabolism

Precisely timed ecdysteroid release is important for the induction of moulting and metamorphosis and requires careful regulation of biosynthesis and inactivation [57]. Several of the phenotypic features and molecular changes that we observed following *spidey* misexpression are consistent with a disruption of ecdysteroid metabolism. First, the significant delay to pupation, presence of malformed forelegs, and failure to metamorphosize observed in *spidey* knockdown flies are reminiscent of other ecdysone-related defects. Second, the early pupal lethality we observed following continuous overexpression of *spidey* is also consistent with previously characterized terminal phenotypes of ecdysone-deficient mutants. Third, the developmental defects induced by *spidey* knockdown were accompanied by an increase in 20HE (but not makisterone A) catabolism in L3 stage. In contrast, *spidey* overexpression led to a substantial decrease in both 20HE and makisterone A levels. Lastly, misexpression of *spidey* alters expression of *Cyp18a1*, a member of the Cytochrome P450 monooxidase family that plays an essential role in ecdysone inactivation [39]. Loss of Cyp18a1 function and its overexpression leads to similar *spidey* misexpression phenotypes, namely lethality during the prepupal or early pupal stage [39,42]. It is possible that *spidey* and *Cyp18a1* directly interact with each other in the same metabolic pathway. Alternatively, changes in ecdysteroid levels caused by *spidey* dysregulation could trigger a change in *Cyp18a1* expression through a feedback circuit [58]. The resulting disrupted balance between 20HE and 20HE-oic acid levels or toxicity from excess 20HE-oic acid likely underlies the failure to transition to metamorphosis. While our genetic manipulations and biochemical measurements strongly indicate that *spidey* has a role in steroid metabolism, ultimately, an enzymatic assay is needed to determine the activity of *spidey* as a steroid dehydrogenase.

We note that females appeared to be more severely affected by the dysregulation of ecdysteroids during larval development. A sex-selective vulnerability to changing levels of 20HE and juvenile hormone-induced pupal wing degeneration has been reported in female moths [59]. Difference in EcR isoform expression or downstream signaling between males and females are postulated to underlie the sexually dimorphic responses to 20HE; however, these mechanisms remain untested. Interestingly, adult female CHC levels were less affected by *spidey* knockdown compared to males. The release of 20HE from other tissues such as the ovaries could potentially compensate for any loss of function from the oenocytes.

### Alternative functions for *spidey*

Previously published findings alluded to the possibility that, based solely on sequence similarity, *spidey* encodes a ketoacyl reductase (KAR), a multi-protein elongase complex necessary for the synthesis of long chain fatty acids (LCFA) and very long chain fatty acids (VLCFA) [36]. We thoroughly investigated this possibility and found no evidence for the role of *spidey* in fatty acyl elongation. If *spidey* is involved in fatty acid elongation, we should have seen overall lower FA levels or an enrichment of shorter chain FAs. However, quantitative measurements of LCFA and VLCFA following knockdown of *spidey* in the oenocytes revealed no significant decrease in FA amounts (S5 Fig). In fact, total FA levels were actually higher in *spidey*^*KD*^ flies at 7 days old. Amounts of methyl branched CHCs, which require elongation of malonyl-CoA and acyl-CoA precursors via KAR [60,61], were also significantly higher following *spidey* knockdown in 8 day old males (S5 Table). Moreover, ubiquitous knockdown of *spidey* throughout the fly also did not change FA levels or FA lengths indicating that *spidey* is not likely to function in FA elongation in other tissues. Lastly, we tested the possibility that *spidey*^*KD*^ flies exhibit a loss of tracheal waterproofing, a distinct phenotype associated with the loss of LCFAs [36]. We placed *spidey*^*KD*^ larvae at 2^nd^ and 3^rd^ instar stages in food stained with blue dye and looked for staining of the tracheal system and hemolymph, indicating loss of waterproofing. No staining was observed in either stage (N = 10, each age; data not presented). Taken together, the loss of *spidey* function did not phenocopy either of the prominent phenotypes associated with long chain fatty acid loss function; therefore, these results do not support the role of *spidey* as a canonical KAR.

### *Spidey* contributes to oenocyte maturation in early adulthood

In Diptera, oenocytes formed during embryonic development disappear during metamorphosis and are replaced during pupal formation by progenitors from a separate population, giving rise to adult oenocytes [62]. Shortly after pupal emergence, *Drosophila* undergo significant muscle and neural tissue remodelling that is triggered by a carefully-timed drop in ecdysteroid levels [63–67]. Our results show that silencing *spidey* expression during this period induced long-lasting effects on oenocyte development and CHC production. Taken together, these observations indicate that adult oenocyte viability is likely to be regulated during the early adult stage via *spidey-*mediated control of ecdysteroid levels. While we did not directly measure levels of 20HE and other ecdysteroids in eclosed adults, the high ratio of 20HE-oic acid to 20HE in 3^rd^ instar larval stage indicates that the unavailability of 20HE due to rapid inactivation is likely to contribute to oenocyte- and CHC-related defects during this early adult window.

### Steroidal maintenance of adult tissues

Silencing *spidey* expression only in the oenocytes resulted in the eventual loss of oenocytes in older flies and a concomitant decrease in cuticular lipid production. The loss of CHCs has dramatic consequences on life history traits including resistance to desiccation, starvation and oxidative stress, and culminates in a shortened lifespan, consistent with previous work [16,28,68]. Could oenocyte viability throughout adulthood depend on ecdysteroid signaling? In larvae, ecdysteroid supplementation across a range of concentrations did not rescue the lethality phenotype (S3 Fig). However, three lines of evidence support a role for 20HE signaling in adult oenocytes. First, our results reveal that 20HE feeding provides a short-term rescue of CHC levels in *spidey*^*KD*^ flies. Second, knockdown of the Ecdysone Receptor gene *EcR* within the oenocytes produced a phenotype consistent with the knockdown of *spidey*: relatively lower levels of (*Z*)-7-tricosene were observed in both cases (S3 Table). Third, previous reports using an ecdysone response element reporter found that adult oenocytes exhibit high levels of EcR / Ultraspiracle (EcR/ USP) reporter activity that increased with age [43]. Taken together, these observations indicate that oenocytes are likely to require 20HE for viability, similar to ovaries in adult females [69].

### Conclusion

Our genetic screen led to the identification of several systemic regulators of pheromone production and revealed a new role for ecdysteroid signaling in adult animals. In addition to insulin-related signaling [13] and juvenile hormone [70], our results show that ecdysone is an essential hormonal regulator of pheromone production. A number of open questions remain about the dynamics and interaction of ecdysteroids and oenocytes. The possible activity of 20HE in other tissues could contribute to the maintenance of oenocyte function. Moreover, it remains to be shown whether the rescue of CHC levels by 20HE feeding takes place directly via EcR/ USP activation in the oenocytes. Lastly, since 20HE feeding resulted in only a partial rescue of CHC synthesis and oenocyte GFP intensity, other molecules are clearly involved. The timing and concentration of 20HE release from the oenocytes might also affect the efficacy of the rescue. Future elucidation of these details will allow us to understand the molecular and cellular mechanisms by which environmental and social conditions shape pheromone production via hormone regulators and, as a consequence, influence behavior, reproduction, and potentially, speciation.

## Materials and Methods

### Fly stocks

The following lines were used: *UAS-RNAi* effector lines were obtained from the Vienna *Drosophila* Resource Centre (see S1 Table for a complete list); *oenocyte-Gal4*, *tubulin-Gal80*^*ts*^ (*oeno-Gal4;* courtesy of J.C. Billeter and J. Levine, University of Toronto, Mississauga, CA) [16]*; doublesex-Gal4 (dsx-Gal4*), *elav-Gal80* (courtesy of S. Goodwin, University of Oxford, UK) [71]; *GeneSwitch-oenocyte-Gal4* (*GSoeno-Gal4)* and *GeneSwitch-tubulin-Gal4* (*GStub-Gal4*; both *GS* lines courtesy of S. Pletcher, University of Michigan, Ann Arbor, USA); *UAS-mCD8-eGFP* (Bloomington *Drosophila* Stock Center, USA); and *UAS-CG1444-3XHA* (*spidey*.*HA;* FlyORF, University of Zurich, CH) [72].

Genetic controls were generated by crossing virgin females of the driver lines to *w1118*. For screening, all crosses were raised at 25°C, separated by sex during the pupal stage, and transferred to 29°C to enhance *Gal4* production. Flies were kept at 29°C for 5–8 days after collection on a 12 hours light/dark cycle.

### Generation of *UAS-CG1444* transgenic line

The ORF of *CG1444* was amplified from cDNA synthesized from 3^rd^ instar larvae RNA (Canton-S) using primers CG1444-ORF-EcoR1-F (5' -ggGAATTCATGGAGGAGAACAACTCGCAAGTGC-3') and CG1444-ORF-XbaI-R (5'-ggTCTAGACTACTGTTCCTTGGCCAGGCGGCGCA-3') and cloned directly into *pWalium10-moe* vector [73] via the *EcoRI* and *XbaI* sites (underlined). Injection of construct into PhiC31-containing attP embryos (Bloomington #3622, targeting chromosome II or Bloomington #24749, targeting chromosome III) and recovery of transgenic individuals was performed by BestGene Inc. (Chino Hills, CA, USA).

### GeneSwitch knockdown

*GSoeno-Gal4* allows conditional activation of the *Gal4* transgene upon exogenous application (by feeding) of RU-486 (11β-(4-Dimethylamino)phenyl-17β-hydroxy-17-(1-propynyl)estra-4,9-dien-3-one) (Sigma-Aldrich, MO, USA) diluted in ethanol. Crosses were raised at 25°C, separated by sex at the pupal stage and fed standard media containing 200 μM of RU-486 upon eclosion. Control flies were raised on standard medium supplemented with ethanol. Flies were kept at 29°C after collection on a 12 hour light/dark cycle and used for assays at 6–8 days old unless otherwise stated.

### *Spidey* overexpression

S*pidey* overexpression lines (*oeno>spidey* and *oeno> spidey*.*HA*) were raised continuously at 19°C or 29°C on standard medium and maintained on a 12 hours light/ dark cycle. Fresh food vials were provided daily.

### Direct Analysis in Real Time mass spectrometry

Six to 10 flies of each genotype were analysed. Following anesthetization on ice, whole flies were held by their wings with fine forceps and placed between the gas inlet and outlet of the Direct Analysis in Real Time (DART) ion source. Cuticular hydrocarbon analysis was performed using a modified version of the method previous described in [74]. Briefly, the DART source was operated in positive ion mode with helium gas with the gas heater set to 200°C. The glow discharge needle potential was set to 3.5 kV. Electrode 1 was set to 150 V, and electrode 2 (grid) was set to 250 V. The mass spectrometer (AccuTOF-DART; JEOL USA, Inc., MA, USA) was run in the positive-ion mode at a resolving power of 6,000 (FWHM definition). Mass spectra were sampled and stored at one spectrum per second with an acquisition range of 60 to 1000 mass to charge ratio (*m/z*). Mass spectrum of an external reference standard, polyethylene glycol, was acquired in each data file for the calibration of mass measurements. In general, protonated molecules ([M+H]^+^) are observed. In contrast to gas chromatography MS (GCMS), saturated hydrocarbons are not detected by DART MS (though under some conditions molecular radical cations may be observed) [75]. Both methods are capable of detecting alkenes, polar, and apolar molecules. However, DART MS is unable to differentiate between isobaric compounds hence compounds with identical molecular weight but double bonds in different positions (*e*.*g*., (*Z*)-7-tricosene and (*Z)-*9-tricosene, appear as the same signal in the DART CHC profile).

The mass-calibrated and centroided mass spectra acquired by MassCenter software (JEOL USA) were exported as text files for processing with Mass Mountaineer software (RBC Software, Portsmouth, NH, available from mass-spec-software.com). Peaks assignment was based on *m/z* values within 0.007 u of the theoretical *m/z* value. To assess changes in the relative abundance of each CHC species, each signal was normalised to the relative abundance of a reference CHC using the following calculation:

For males,

$$relative\;CHC\;intensity=\;\left(\frac{CHC\;value}{tricosene\;value}\right)\times 100$$

For females,

$$relative\;CHC\;intensity=\;\left(\frac{CHC\;value}{heptacosadiene\;value}\right)\times 100$$

Male pheromones were further categorised into monoenes, mono-oxygenated, di-oxygenated and tri-oxygenated species and the average of CHC species from all replicates in each category were summed. Candidate lines with CHC profile differences were selected based on normalized values that were 2 standard deviations above or below the pooled average. GCMS samples were prepared by incubating 8 cold anaesthetised flies of each genotype at room temperature for 20 minutes with 120 μL of hexane containing 10 μg/mL hexacosane as an internal standard. 100 μL of the extract was transferred into a fresh glass vial and allowed to evaporate at room temperature. Samples were stored at -20°C. Three replicates were prepared for each genotype.

### Gas chromatography mass spectrometry

Analysis by GCMS was performed on two systems. A QP2010 system (Shimadzu, Kyoto, Japan) equipped with a DB-5 column (***5***%-Phenyl-methylpolysiloxane column; 30 m length, 0.25 mm ID, 0.25 μm film thickness; Agilent) was used. Ionization was achieved by electron ionization (EI) at 70 eV. One microliter of the sample was injected using a splitless injector. The helium flow was set at 1.9 mL/min. The column temperature program began at 50°C, increased to 210°C at a rate of 35°C /min, then increased to 280°C at a rate of 3°C/min. A mass spectrometer was set to unit mass resolution and 3 scans/ sec, from *m/z* 37 to 700. Chromatograms and mass spectra were analysed using GCMSsolution software (Shimadzu). In addition, a SCION GCMS (Bruker Corp., Billerica, USA) with a BPx-1701 column (60 m x 0.25 mm, x 0.10 μm; Restek Corp., Bellefonte, USA) was used for fatty acid measurements of *GStub>CG1444-RNAi* adults. The helium flow was set at 1.5 mL/min. The column temperature began at 45°C for 4 min, increased to 180°C at a rate of 20°C/min and held at 180°C for 4 min, then increased to 280°C at a rate of 3°C/ min and held for 30 min. Chromatograms and mass spectra were analysed using MS Workstation software (Bruker)

The relative abundance of each CHC is calculated by dividing the area under the peak by the total area of all peaks detected in the chromatogram. The relative abundance of CHCs from each transgenic line was then compared with that of the control with a Student’s two-tailed t-test (GraphPad Prism 5, Graph Pad Software Inc., CA, USA). For total CHC levels, the area under each of the CHC peaks were summed and normalized to the area under the peak for the spiked hexacosane standard.

### Fatty acid quantification

Fatty acid extraction was modified from [76]. Five to nine flies were homogenised in 100 μL of PBS followed by an addition of 600 μL of chloroform: methanol (1:2, v/v) containing 10 μg/ mL hexacosane or 10 μg/ mL pentadecanoic acid (Sigma-Aldrich). The mixture was vortexed for 1 minute before being shaken at high speed at 4°C for 2 hours. After which, 200 μL of chloroform and 250 μL of water was added to the mixture and vortexed for 1 minute. The samples were then centrifuged at 7500 x g RCF for 2 minutes for phase separation and the lower organic phase was collected. The extraction was repeated twice by adding 400 μL of chloroform to the remaining aqueous phase. The organic extracts were pooled and concentrated by N_2_ evaporation.

The extracts were esterified with the addition of 0.5N Methanolic HCl (Sigma-Aldrich) and incubation in a water bath at 65°C for 1.5 hours with occasional vortexing. The solvents were evaporated under N_2_ and the samples stored at -20°C until reconstitution with hexane prior to analysis. Peaks corresponding to FA methyl ester (FAME) were identified based on retention time and presence of diagnostic ion m/z 74. The relative abundance of each species was calculated by dividing the area under each peak by the internal standard. Total and individual FA abundances were compared with a Student’s two-tailed t-test (GraphPad Prism 5, Graph Pad Software Inc., CA, USA).

Quantification of ecdysteroids in larvae *Spidey*^*KD*^ *and spidey*^*control*^ larvae were staged by collecting embryos on grape juice agar for intervals of 1.5 hours. Embryos were washed with PBS containing 0.05% TritonX-100 then bleached for 30 seconds in 50% sodium hypochlorite followed by 2 washes with distilled water. The embryos were then placed individually into 2 mL polypropylene tubes containing 0.5 mL of standard food with or without 200 μM RU-486. For *oeno>spidey* and *oeno>spidey*.*HA* experiments, crosses were raised continuously at either 19 or 29°C and parental lines were flipped into fresh food vials every 24 hrs. L1 [collected 40 hours after egg laying (AEL)], L2 (collected 72 hours AEL for controls and 96 hours AEL for *spidey*^*KD*^ to account for delayed maturation; presence of mouthhooks and size were also used as criteria for stage), L3 wandering larvae (collected 4 hours before pupariation, approximately 1–2 hours after emerging from food and becoming inactive on the walls) and pupae (40 hours after pupation) were collected into 1.5 mL polypropylene tubes, placed in liquid nitrogen for 10 sec and stored at -80°C prior to analysis.

Steroid quantification was performed as previously described in [77]. Frozen animals were smashed in 1.5 mL plastic tubes (Eppendorf, Hamburg, Germany) extracted with 1 mL of methanol overnight. Then samples were centrifuged for 5 min at 13400 rpm and the supernatant was collected. The residual pellet was twice re-extracted with 1 mL of methanol. The combined extracts were dried down in a vacuum concentrator, re-dissolved in 1 mL of methanol containing 0.25 pmol of muristerone A internal standard and twice extracted with 3 mL of hexane to remove the bulk of di- and triacylglycerols and sterols. The collected lower methanol fraction was dried and re-dissolved in 180 μL of 70% aqueous methanol. Samples were loaded on C18 MicroSpin columns (Nest group, Southborough, MA, USA). Columns were twice washed with 180 μL of 70% methanol to remove the bulk of glycerophospholipids and centrifuged for 1 min at 2000 rpm. Eluates were dried down, re-dissolved in 15% aqueous methanol and transferred to 150 μL plastic vials. HPLC was performed on Agilent 1200 system equipped with a trap column (OPTI-PAK, 1 μL, C18) from Dichrom GmbH (Marl, Germany) that was mounted in-line to a 0.5 mm × 150 mm analytical column packed with Zorbax SB-C18 5 μm (Böblingen, Germany). The mobile phase consisted of solvent A (0.1% aqueous formic acid) and solvent B (0.1% formic acid in neat acetonitrile). The gradient elution program was as follows: delivering 5% of B during first 10 min until the sample is loaded and concentrated on the trap column; ramping from 15% to 30% of B between 11 min to 30 min; increasing up to 100% of B in 1 min and holding for 9 min; stepping down to 5% of B in 1 min and holding for 19 min to equilibrate the column to starting conditions. The flow rate was 10 μL/ min; injection volumes are specified for each experiment.

MS spectra were acquired in *t-*SIM mode on a Q Exactive tandem mass spectrometer (Thermo Fisher Scientific, Waltham, MA, USA). Acquisition settings were set for mass resolution R_m/z = 200_ 700000; automated gain control target 10^5^; maximum injection time of 200 ms; the width of transmission window of the analytical quadrupole of 1 Da. Extracted ion chromatograms were produced for [M+H]^+^ molecular ions of ecdysteroids assuming 2 ppm mass accuracy and their quantification was performed using Xcalibur 2.2 software. Ecdysteroid quantities were log transformed and analysed using a two-tailed Student’s t-test (GraphPad Prism 5).

Additional measurements of ecdysteroids in *oeno>spidey*.*HA* larvae were performed on a different chromatography set up. 25 μL of standard or extract was injected onto a hypersil gold C18 column (50 x 2.1 mm, 1.9 um, Thermo Scientific Inc.) and separated with a mobile phase consisting of 0.1% formic acid in water (solvent A) and 0.1% formic acid in methanol (solvent B) at a flow rate of 350 μL/min with the following gradient: from 0 to 2 min, 90:10 to 80:20; from 2 to 8 min, 80:20 to 20:80; hold at 20:80 for 2 min, then back to the initial condition of 90:10 and equilibrate for 5 min). A seven-point calibration curve was prepared covering a range of 0.05 ~ 500 ng/ mL. The limit of detection for the analytes is from 1 to 20 pg/ mL. Ecdsyteroid standards were obtained from Santa Cruz Biotechnology, Inc. (Dallas, USA).

### Quantitative analysis of mRNA transcripts

Total RNA of whole flies (5 whole flies per replicate) or larvae (5 larvae per replicate) was prepared using TRIzol Reagent as per the manufacturer’s instructions. The extracted RNA was treated with TURBO DNA-*free* Kit (AM1907, Life Technologies, USA) prior to reverse transcription reaction using Superscript III Reverse transcriptase (18080–044, Life Technologies, USA) to obtain cDNA. Five ng of cDNA was used for qPCR, using KAPA SYBR FAST qPCR Kit (KK4604, KAPA Biosystems, USA) and Applied Biosystems 7900HT Fast Real-Time PCR system. The conditions used were as recommended in the KAPA SYBR FAST qPCR Kit. Relative transcript levels were calculated as 2^ΔCt^ [78], ΔCt = Ct_rp49_—Ct_sample_. Three technical replicates were performed for each genotype, with 3 biological replicates. The following primer sequences were used: *spidey* forward, 5’- TTCTACCCGGCATGATTAGC; *spidey* reverse, 5’- GCTCCTTGTACTCCGTCTGC; *Cyp18a1 forward*, *5’-* TACCTGCCCATTACCGAGTC*; Cyp18a1 reverse*, *5’-* ACCCATTGAGTTCCACATCC*; rp49* forward, 5’-ATCTCCTTGCGCTTCTTGG; *rp49* reverse, 5’-CAAGCCCAAGGGTATCGAC.

### Electron microscopy

Electron micrographs of freshly dissected legs were obtained on a JEOL JSM-6360LV scanning electron microscope at 20 kV (JEOL, Peabody, MA).

### Fly survival assay

Embryos were collected on grape juice agar for 6 hours and placed in vials containing standard media. Each vial was transferred to restrictive temperature of 29° in 24 hour intervals after egg laying (AEL). The number of intact flies per vial was counted 11 days AEL and the percentage survival was calculated by dividing the number of intact flies by the number of pupae formed.

### Supplement feeding assay

Embryos were collected on grape juice agar and transferred to standard media containing various supplements and kept at 25°C until pupal stage, then separated by sex and transferred to 29°C at 10 days AEL. The number of intact flies was counted 5 days after adult eclosion. The final concentrations of palmitic acid (C16:0), stearic acid (C18:0), oleic acid (C18:1), and linoleic acid (C18:2) were at 50 or 500 μg/ mL. Concentrations for other supplements are as follow: behenic acid (C22:0), 50 μg/ mL; 20-hydroxyecdysone (20HE), 7.2, 0.72, or 0.072 μg/ mL; and cholesterol, 0.14 μg/ mL. For adult ecdysone rescue experiments, food was supplemented with 8.33 μg/ mL 20HE. All chemicals were acquired from Sigma-Aldrich.

### Adult longevity assay

Ten flies were placed in vials containing standard food or standard food with 200 μM RU-486. Flies were maintained at 29°C on a 12 hour light/dark cycle and transferred into fresh media every 3 to 4 days. The number of dead flies was counted every day. Ten replicates were established for each treatment.

### Starvation assays

10 flies were transferred to 25 mL vials containing 10 mL of 1% agar or 1% agar with 20 μM RU-486. The flies were maintained at 29°C and the number of dead flies was counted every 2 hours. Five replicates were established for each treatment.

### Desiccation assay

Groups of 10 flies were transferred into an empty 25 mL vial and confined to the lower half of the vial with a foam stopper with approximately 3 g of Drierite dessicant (Sigma-Aldrich) in the top half. The vial was sealed with Parafilm and maintained at 25°C. The number of dead flies was recorded every hour. Five replicates were established for each treatment.

### Oxidative stress assay

Flies were starved for 6 hours in empty vials in groups of 10 and transferred to 25 mL vials containing 10 mL of 0.8% low melt agarose and 10% sucrose in phosphate buffered saline (pH 7.4) containing 20 mM paraquat (Sigma-Aldrich), with or without 200 μM of RU-486. Ten replicates were established for each treatment.

### Spider-Man assay

Genetic crosses and control lines were raised at 25°C and switched to 29°C at late pupal stage. Flies were flipped onto new food every 5–7 days and dead flies counted every 2–3 days. The sticky Spider-Man phenotype is defined as flies that are found dead on the wall of the vial (rather than dead on the surface of the food) and do not detach despite vigorous shaking and knocking.

### Oenocyte quantification

Dorsal abdominal fillets were prepared according to [79]. Fluorescent images were obtained with a Leica MZ10F fluorescence stereomicroscope fitted with Nikon DXM 1200F camera and Nikon ACT-1 version 2.10 imaging software. Fluorescent images were first converted to an 8-bit grayscale image and segmented with the pre-set default threshold setting using ImageJ. The total area occupied by GFP signal within a 9 unit square area consisting of at least 3 bands of oenocytes spanning the medial line was measured.

## Supporting Information

S1 Fig Expression pattern of *oeno-Gal4* and *dsx-Gal4* drivers used for screening. Both the *oeno-Gal4* (**A**) and *dsx-Gal4* (**B**) drivers exhibit robust expression in the oenocytes of males and females (arrowheads). The *oeno-Gal4* driver also labels the male accessory glands (**A**; arrowheads) whereas *dsx-Gal4* expression is found in the seminal vesicles and ejaculatory bulb (**B**; arrowheads). See S6 Table for full genotypes.
(PDF)

S2 Fig Representative DART MS spectra of transgenic females exhibiting changes in cuticular lipid profiles. (**A**) Suppression of the desaturase *desat1*in the oenocytes (red spectrum) resulted in overall lower signal intensity, elevated levels of monoenes relative to dienes and an increase in oxygenated species (arrows) relative to genetic controls (blue spectrum). See S6 Table for full genotypes. (**B**) Suppression of the desaturase *deastF* in the oenocytes (red spectrum) resulted in a decrease of female-specific dienes (C27:2 and C29:2) and an increase in monoenes of the corresponding chain length (arrows) relative to genetic controls (blue spectrum). (**C**) Suppression of the elongase *eloF* in the oenocytes (red spectrum) resulted in an overall shortening of long chain dienes, very low levels of C27:2 and C29:2, and higher levels of C25:2 relative to genetic controls (blue spectrum).
(PDF)

S3 Fig Sterol and fatty acid supplementation does not rescue female pharate lethality of *oeno>spidey*^*RNAi*^ flies. (**A**) Supplementation with 20-hydroxyecdysone (20HE) or cholesterol; Fisher’s exact test, not significant, N = 16–47. See S6 Table for full genotypes. (**B**) Supplementation with palmitic acid (C16:0), stearic acid (C18:0), oleic acid (C18:1) or linoleic acid (C18:2); Fisher’s exact test, not significant, N = 33–51.
(PDF)

S4 Fig *Spidey* expression in adult animals. *Spidey* levels in wildtype Canton-S flies did not significantly differ throughout adulthood (one-way ANOVA, not significant). Expression levels at 7 and 15 days old were normalized to levels at 1 day old. Data represent the mean of 3 experimental replicates ± SEM. *Rp49* was used as an internal control for normalization. See S6 Table for full genotypes.
(PDF)

S5 Fig Fatty acid quantities following *spidey* knockdown. (**A**) Levels of total fatty acids (FA) *spidey*^*KD*^ female flies were significantly higher than age-matched controls at 7 and 15 days old. (**B**) Total FA levels in 7 day old *spidey*^*KD*^ male flies were higher than in age-matched controls. Differences in FA levels at other ages were not detected. Each data point represents the average of 3 experimental replicates; one-way ANOVA with post-hoc Holm-Šidák test. See S6 Table for full genotypes. (**C**) Following ubiquitous knockdown of *spidey* using a *GStub-Gal4* driver, no significant differences in total FA levels were observed between *spidey*^*tubKD*^ and *spidey*^*tubcontrol*^ male flies; one way ANOVA. (**D**) No significant differences in the amounts of individual FA species were observed between *spidey*^*tubKD*^ and *spidey*^*tubcontrol*^ male flies; one way ANOVA.
(PDF)

S1 Movie *Oeno>spidey*^*RNAi*^ flies exhibiting the Spider-Man “sticky” phenotype adhere to the walls of the vial and cannot be dislodged by vigorous shaking or tapping. (MP4)

S1 Table RNAi lines used in screen. (DOCX)

S2 Table DART MS quantification of cuticular lipid profiles from female transgenic lines. (DOCX)

S3 Table DART MS quantification of cuticular lipid profiles from male transgenic lines. (DOCX)

S4 Table GCMS quantification of CHC profiles from female transgenic lines. (DOCX)

S5 Table GCMS quantification of CHC profiles from male transgenic lines. (DOCX)

S6 Table Full genotypes shown in each figure. (DOCX)
