# Supplementary figures and images for: Steroid Hormone Signaling Is Essential for Pheromone Production and Oenocyte Survival
Source: PLoS Genet. 2016 Jun 22;12(6):e1006126. doi: 10.1371/journal.pgen.1006126 (PMC4917198; doi:10.1371/journal.pgen.1006126)

# **A** *oeno>GFP*

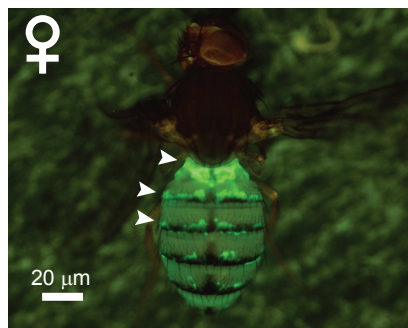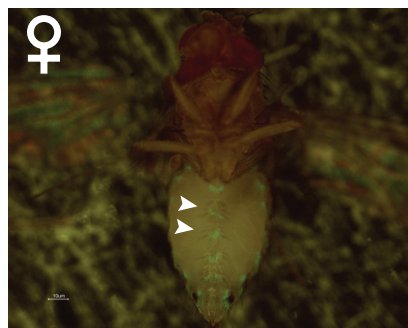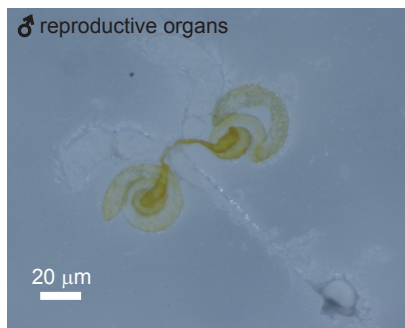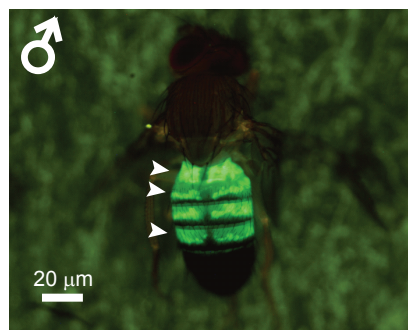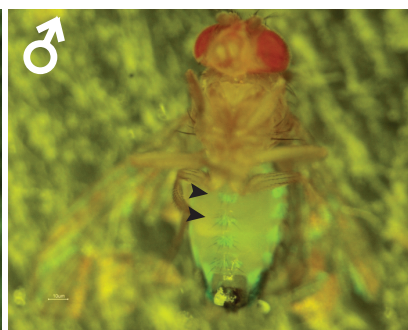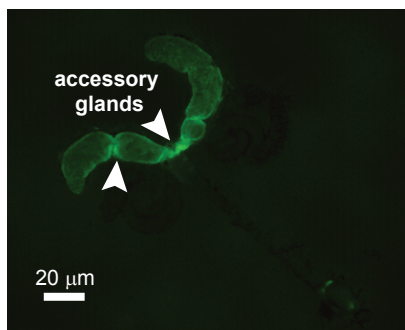

# **B** *dsx>GFP*

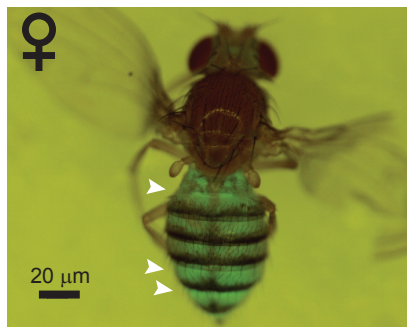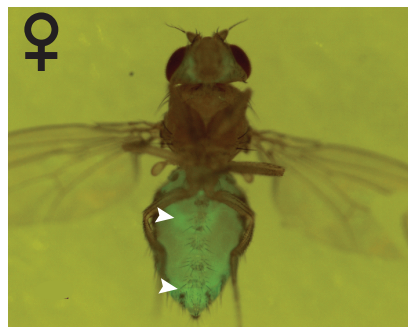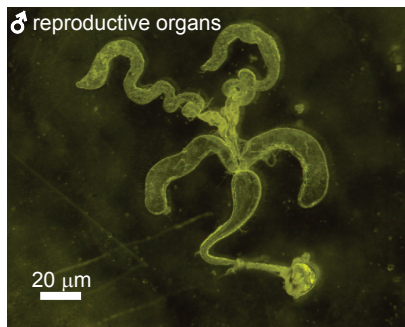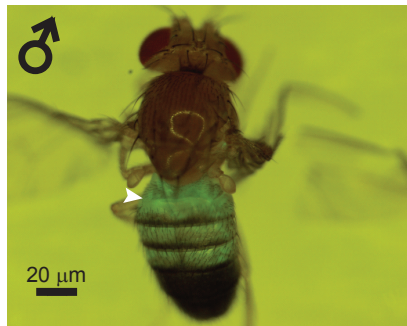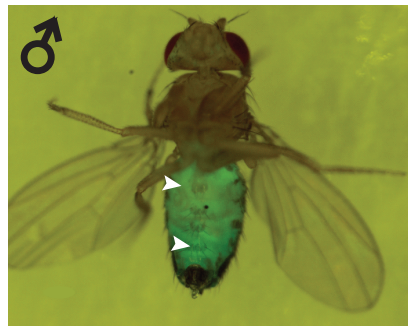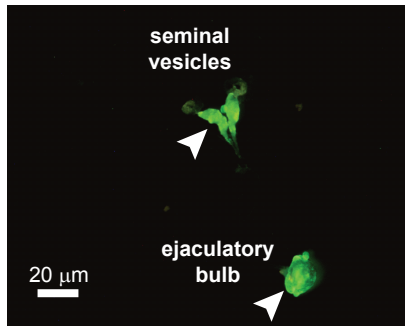

Supplement: S1 Fig — Both the oeno-Gal4 (A) and dsx-Gal4 (B) drivers exhibit robust expression in the oenocytes of males and females (arrowheads). The oeno-Gal4 driver also labels the male accessory glands (A; arrowheads) whereas dsx-Gal4 expression is found in the seminal vesicles and ejaculatory bulb (B; arrowheads). See S6 Table for full genotypes. (PDF) [file pgen.1006126.s001.pdf]

**A**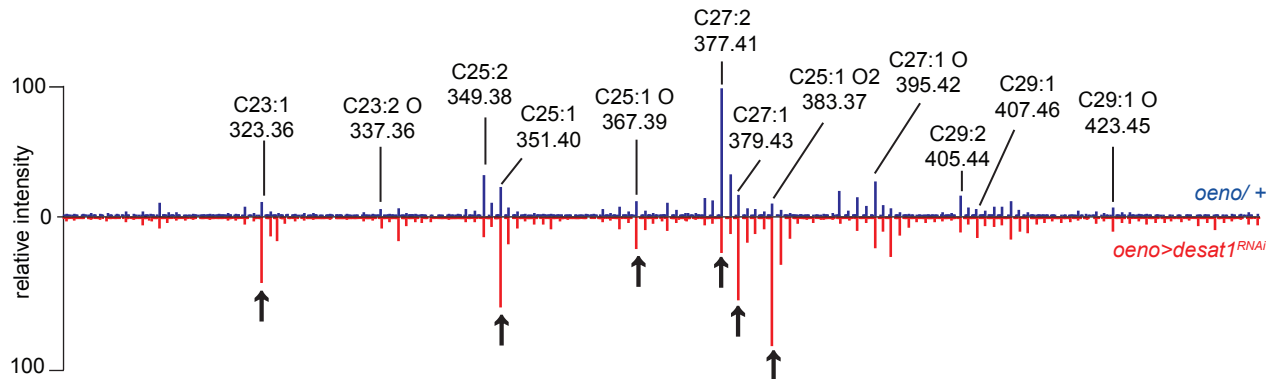**B**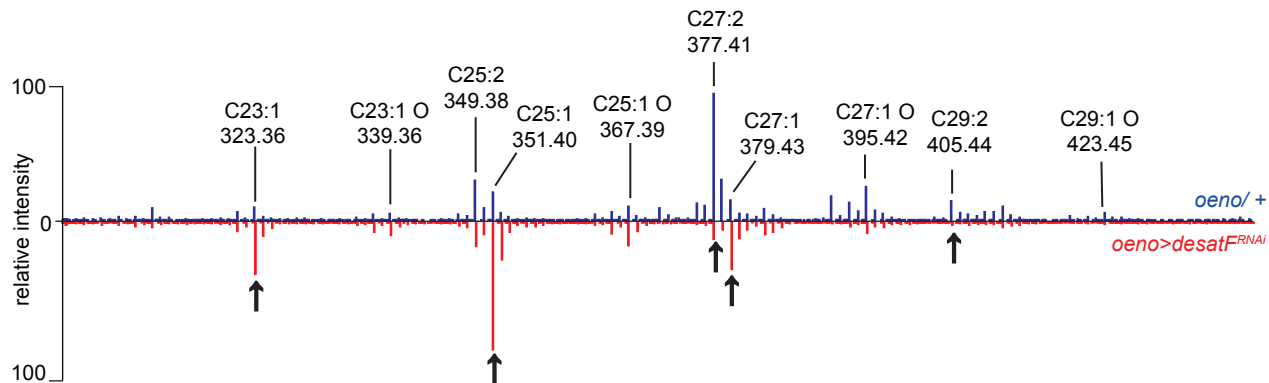**C**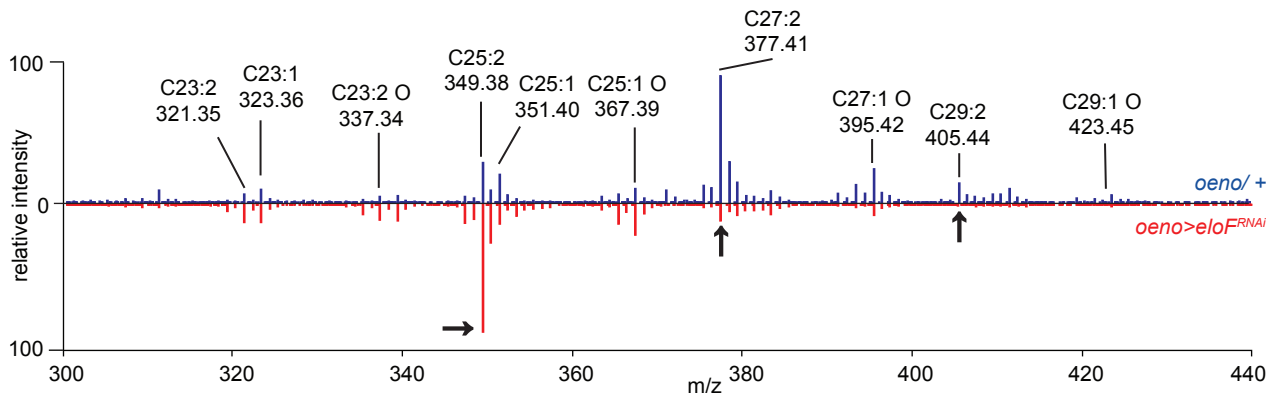

Supplement: S2 Fig — (A) Suppression of the desaturase desat1in the oenocytes (red spectrum) resulted in overall lower signal intensity, elevated levels of monoenes relative to dienes and an increase in oxygenated species (arrows) relative to genetic controls (blue spectrum). See S6 Table for full genotypes. (B) Suppression of the desaturase deastF in the oenocytes (red spectrum) resulted in a decrease of female-specific dienes (C27:2 and C29:2) and an increase in monoenes of the corresponding chain length (arrows) relative to genetic controls (blue spectrum). (C) Suppression of the elongase eloF in the oenocytes (red spectrum) resulted in an overall shortening of long chain dienes, very low levels of C27:2 and C29:2, and higher levels of C25:2 relative to genetic controls (blue spectrum). (PDF) [file pgen.1006126.s002.pdf]

**A**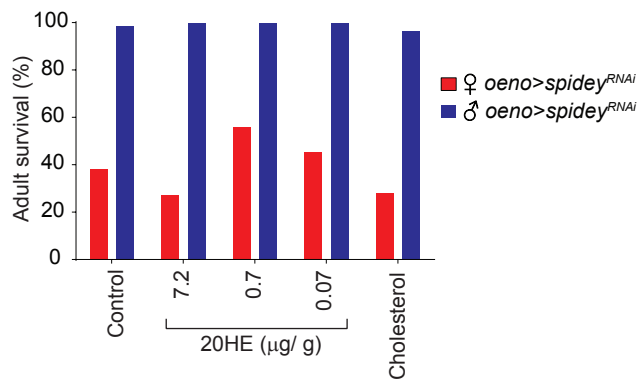**B**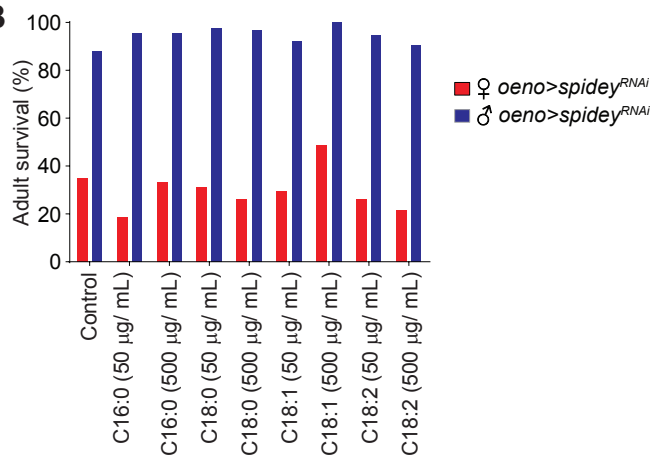

Supplement: S3 Fig — (A) Supplementation with 20-hydroxyecdysone (20HE) or cholesterol; Fisher’s exact test, not significant, N = 16–47. See S6 Table for full genotypes. (B) Supplementation with palmitic acid (C16:0), stearic acid (C18:0), oleic acid (C18:1) or linoleic acid (C18:2); Fisher’s exact test, not significant, N = 33–51. (PDF) [file pgen.1006126.s003.pdf]

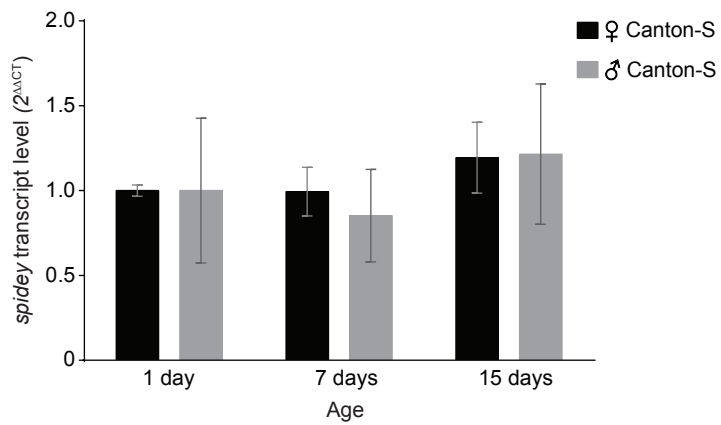

Supplement: S4 Fig — Spidey levels in wildtype Canton-S flies did not significantly differ throughout adulthood (one-way ANOVA, not significant). Expression levels at 7 and 15 days old were normalized to levels at 1 day old. Data represent the mean of 3 experimental replicates ± SEM. Rp49 was used as an internal control for normalization. See S6 Table for full genotypes. (PDF) [file pgen.1006126.s004.pdf]

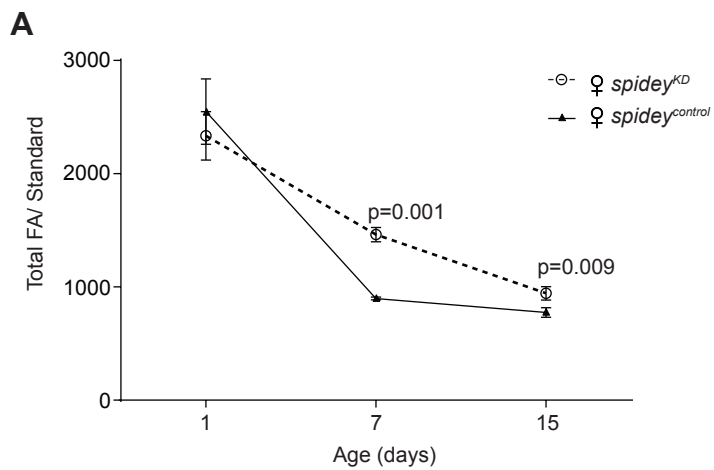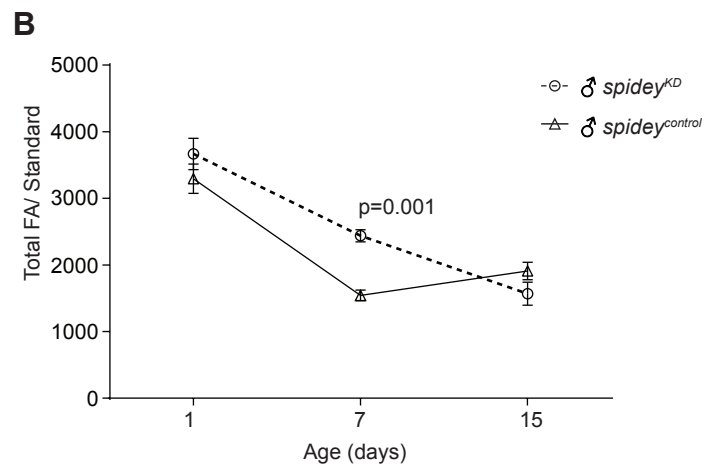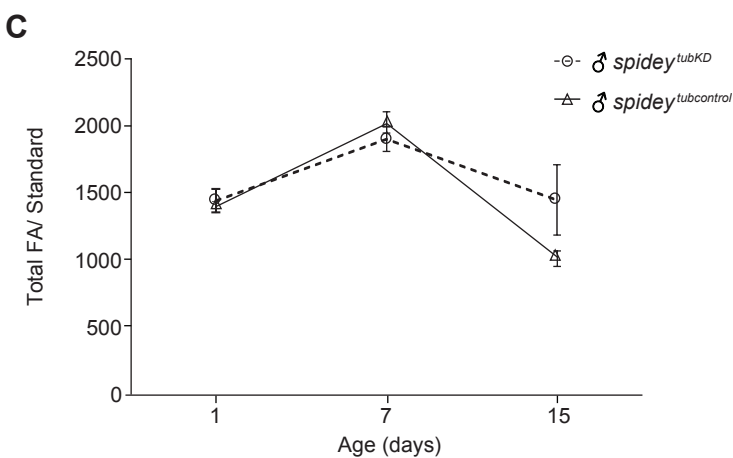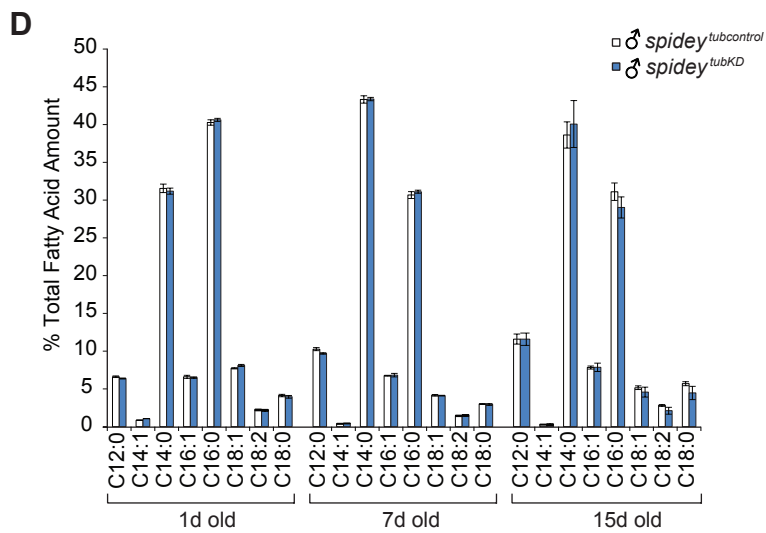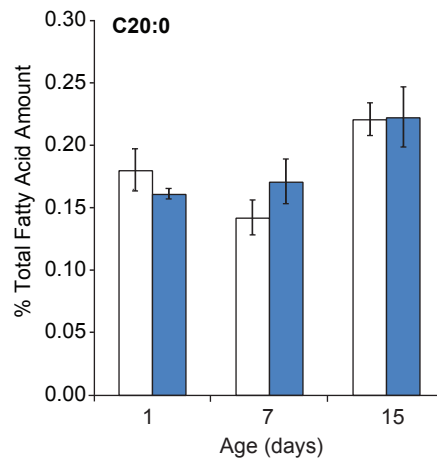

Supplement: S5 Fig — (A) Levels of total fatty acids (FA) spideyKD female flies were significantly higher than age-matched controls at 7 and 15 days old. (B) Total FA levels in 7 day old spideyKD male flies were higher than in age-matched controls. Differences in FA levels at other ages were not detected. Each data point represents the average of 3 experimental replicates; one-way ANOVA with post-hoc Holm-Šidák test. See S6 Table for full genotypes. (C) Following ubiquitous knockdown of spidey using a GStub-Gal4 driver, no significant differences in total FA levels were observed between spideytubKD and spideytubcontrol male flies; one way ANOVA. (D) No significant differences in the amounts of individual FA species were observed between spideytubKD and spideytubcontrol male flies; one way ANOVA. (PDF) [file pgen.1006126.s005.pdf]
