# Supplementary material for: Steroid Hormone Signaling Is Essential for Pheromone Production and Oenocyte Survival
Source: PLoS Genet. 2016 Jun 22;12(6):e1006126. doi: 10.1371/journal.pgen.1006126 (PMC4917198; doi:10.1371/journal.pgen.1006126)
Supplement: S1 Table — (DOCX) [file pgen.1006126.s007.docx]

**Supplemental Table 1**. RNAi lines used in screening.

| **Predicted function^1^** | **CG Number^2^** | **Gene name** | **Source^3^** |
| --- | --- | --- | --- |
| Fatty acid elongase | CG32072 | *Elo68α* | VDRC 9206 |
|  | CG16905 | *eloF* | VDRC 48663 |
|  | *CG2781 |  | VDRC 48138 |
|  | CG7842 | *beg* | VDRC 46334 |
|  | CG3523 |  | VDRC 108339 |
|  | CG12170 |  | VDRC 31673 |
| Fatty acid elongation | CG31141 |  | VDRC 29537 |
|  | CG3971 | *Baldspot* | VDRC 30205 |
|  | CG9458 |  | VDRC 48702 |
|  | CG9459 |  | VDRC 48905 |
|  | CG16904 |  | VDRC 103434 |
|  | CG8534 |  | VDRC 49892 |
|  | CG5326 |  | VDRC 47681 |
|  | CG5278 |  | VDRC 43091 |
|  | CG6660 |  | VDRC 6836 |
| Fatty acid transport | *CG7400 | *Fatp* | VDRC 9406 |
|  | CG4830 |  | VDRC 21635 |
|  | *CG6300 |  | VDRC 27576 |
|  | CG11391 |  | VDRC 38495 |
|  | CG11659 |  | VDRC 16258 |
| Very-long-chain fatty acid metabolism | CG31522 |  | VDRC 37329 |
|  | CG3311 |  | VDRC 29689 |
| Pheromone biosynthesis | CG6917 | *Est-6* | VDRC 35607 |
| Regulation of lipid biosynthesis | CG17673 | *Acp70A* | VDRC 52425 |
|  | CG30420 | *Atf-2* | Bloomington 26210 |
|  | *CG8522 | *HLH106* | VDRC 37641 |
|  | *CG11140 |  | VDRC 37726 |
| Long-chain-fatty-acid-CoA ligase | CG4501 | *bgm* | VDRC 34853 |
|  | *CG3961 |  | VDRC 37306 |
|  | CG4500 |  | VDRC 34852 |
|  | CG6178 |  | VDRC 1172 |
|  | CG9009 |  | VDRC 12016 |
|  | CG18155 |  | VDRC 25297 |
| Adult fat body expression | CG10079 | *Egfr* | VDRC 43268 |
|  | CG7266 | *Eip71CD* | VDRC 26009 |
|  | CG18350 | *Sxl* | VDRC 1547 |
|  | CG6824 | *ovo* | VDRC 41584 |
|  | CG1028 | *Antp17* | VDRC 101774 |
|  | CG11648 | *iab6* | VDRC 12024 |
|  | CG5714 | *ecd* | VDRC 28398 |
|  | CG9097 | *bab1* | VDRC 50286 |
|  | *CG9102 | *bab2* | VDRC 49042 |
|  | CG5887 | *desat1* | VDRC 33338 |
|  | CG5887 | *desat1* | VDRC 47141 |
|  | CG7923 | *desatF* | VDRC 7492 |
|  | CG5925 | *desat2* | VDRC 103666 |
|  | CG9434 | *Fst* | VDRC 39070 |
| **Predicted function^1^** | **CG Number** | **Gene name** | **Source^2^** |
| Lipid modification | *CG5162 |  | VDRC 14661 |
|  | CG14034 |  | VDRC 100114 |
|  | CG17097 |  | VDRC 41244 |
|  | CG18258 |  | VDRC 20621 |
|  | CG31872 |  | VDRC 31028 |
|  | CG10849 | *Sc2* | VDRC 7480 |
|  | CG5137 | *Cyp312a1* | VDRC 44857 |
|  | CG5137 | *Cyp312a1* | VDRC 44858 |
|  | CG5138 | *Cyp312a2* | VDRC 41931 |
|  | CG18609 |  | VDRC 4994 |
|  | CG9743 |  | VDRC 108185 |
|  | CG17292 | *TGL* | VDRC 46538 |
|  | CG7920 | *4-hydroxybutyrate CoA-transferase* | VDRC 21577 |
|  | CG12262 | *Acyl-CoA-dehydrogenase* | VDRC 15053 |
|  | CG14615 | *Acyl-CoA-acyltransferase* | VDRC 24872 |
|  | CG13279 |  | VDRC 42474 |
| Peroxisomal proteins | CG4663 | *Pex13* | VDRC 108829  VDRC 39544 |
|  | CG6871 | *Catalase* | VDRC 6283 |
|  | CG9527 |  | VDRC 24054 |
|  | CG11151 |  | VDRC 107530  VDRC 18129 |
|  | CG12428 | *COT* | VDRC 108291 |
|  | *CG1444 |  | RNAi^(1)^: VDRC 40949 |
|  | *CG1444 |  | RNAi^(2)^: Bloomington 36607 |
|  | *CG17562 |  | VDRC 37365 |
|  | CG18031 |  | VDRC 30220 |
| Proteolysis | CG6289 | *Serpin 77Bc* | VDRC 105515  VDRC 14054 |
| Catalysis | CG2668 | *Peb* | VDRC 18973 |
| LDL receptor family | CG31094 | *LpR1* | VDRC 14756 |
|  | CG31092 | *LpR2* | VDRC 25684 |
| Heme synthetic enzyme (oenocytes) | CG10335 | *Pbgs* | VDRC 40612 |
| Nuclear hormone receptor | *CG1765 | *EcR-A* | VDRC 37058 |
|  | CG8127 | *Eip75B* | VDRC 44851 |
|  | CG9310 | *Hnf-4* | VDRC 12692 |
|  | *CG11502 | *svp* | VDRC 37087 |
| Neuropeptide hormone activity | CG1171 | *Adipokinetic hormone* | VDRC 105063 |
| Stearoyl-coA 9-desaturase | CG15531 |  | VDRC 1397 |

^1^Based on Gene Ontology term.

^2^ *: knockdown resulted in significant change to cuticular lipid profile.

^3^VDRC: Vienna Drosophila Resource Center; Bloomington: Bloomington Drosophila Stock Center.
