# Supplementary material for: Steroid Hormone Signaling Is Essential for Pheromone Production and Oenocyte Survival
Source: PLoS Genet. 2016 Jun 22;12(6):e1006126. doi: 10.1371/journal.pgen.1006126 (PMC4917198; doi:10.1371/journal.pgen.1006126)
Supplement: S2 Table — (DOCX) [file pgen.1006126.s008.docx]

**Supplementary Table 2**. DART MS analysis of female flies from transgenic lines exhibiting significant changes in cuticular lipid profile.

|  | **Signal intensity^1^** | | | | | | | | |
| --- | --- | --- | --- | --- | --- | --- | --- | --- | --- |
| CHC species^2^ | **Pooled Average^3^** | ***oeno>CG7400***  **(N=16)** | ***oeno>CG9102***  **(N=15)** | ***oeno>CG11502***  **(N=16)** | ***oeno*>*CG17562***  **(N=15)** | **Pooled Average^4^** | ***dsx>CG1444* (N=15)** | ***dsx>CG2781* (N=20)** | ***dsx>CG6300* (N=15)** |
| C23:1 | 10.38±3.69 | 11.25±1.24 | 10.88±0.99 | 8.07±0.96 | 9.75±0.87 | 9.80±3.40 | 23.45±1.71* | 1.95±0.61* | 11.95±1.12 |
| C25:2 | 24.93±5.05 | 20.94±0.72 | 13.28±1.07 | 35.40±2.02* | 19.09±0.75 | 14.77±3.05 | 39.43±1.39* | 10.25±0.37 | 14.30±0.55 |
| C27:2 | 100.00±0.00 | 100.00±0.00 | 100.00±0.00 | 100.00±0.00 | 100.00±0.00 | 100.00±0.00 | 100.00±0.00 | 100.00±0.00 | 100.00±0.00 |
| C29:2 | 34.80±12.68 | 66.89±3.52* | 88.63±3.42* | 22.74±1.10 | 94.76±6.13* | 70.73±23.58 | 99.38±3.49 | 70.83±1.65 | 124.30±5.42* |

^1^Values represent mean ± SEM; one way ANOVA with post-hoc Tukey’s HSD, *p<0.05 when compared to pooled average; DART –MS is not able to distinguish between isobaric molecules, hence values for each CHC species represent the sum intensity of all stereoisomers.

^2^Elemental composition is represented by the carbon chain length followed by the number of double bonds.

**^3^**The average signal intensity calculated from of all *oeno-Gal4* transgenic lines.

**^4^**The average signal intensity calculated from of all *dsx-Gal4* transgenic lines.
