# Supplementary material for: Steroid Hormone Signaling Is Essential for Pheromone Production and Oenocyte Survival
Source: PLoS Genet. 2016 Jun 22;12(6):e1006126. doi: 10.1371/journal.pgen.1006126 (PMC4917198; doi:10.1371/journal.pgen.1006126)
Supplement: S3 Table — (DOCX) [file pgen.1006126.s009.docx]

**Supplementary Table 3**. DART MS analysis of male flies from transgenic lines exhibiting significant changes in cuticular lipid profile.

|  | **Signal intensity^1^** | | | | | | | | | | | |
| --- | --- | --- | --- | --- | --- | --- | --- | --- | --- | --- | --- | --- |
| Cuticular lipid species^2^ | **Pooled Average^2^** | ***oeno>***  ***CG1444* (N=15)** | ***oeno>***  ***CG2781* (N=16)** | ***oeno>***  ***CG5162* (N=15)** | ***oeno>***  ***CG7400* (N=19)** | ***oeno>***  ***CG8522* (N=19)** | ***oeno>***  ***CG3961* (N=15)** | **Pooled Average^3^** | ***dsx>***  ***CG1765* (N=16)** | ***dsx>***  ***CG2781* (N=22)** | ***dsx>***  ***CG7400* (N=16)** | ***dsx>***  ***CG11140* (N=15)** |
| Monoenes:  C23:1, C25:1, C27:1, C29:1 | 154.63  ± 14.80 | 194.41  ± 6.21* | 153.88  ± 2.14 | 161.98  ± 4.62 | 226.53  ± 15.27* | 190.73  ± 5.66* | 171.75  ± 5.61 | 181.11  ± 17.00 | 228.38  ± 7.59* | 167.73  ± 4.92 | 252.41  ± 12.55* | 142.43  ± 4.33* |
| Mono-oxygenated:  C_23_H_46_O  C_25_H_48_O | 99.48  ± 27.55 | 134.06  ± 10.58 | 116.97  ± 7.46 | 107.79  ± 14.02 | 124.18  ± 17.14 | 104.69  ± 8.91 | 126.76  ± 15.55 | 112.59  ± 26.88 | 137.88  ± 10.28 | 111.29  ± 6.48 | 145.32  ± 20.45 | 64.52  ± 11.33 |
| Di-oxygenated:  C_23_H_44_O_2_ | 49.29  ± 21.04 | 31.03  ± 4.58 | 116.64  ± 17.46* | 106.86  ± 13.49* | 50.58  ± 11.77 | 47.69  ± 5.57 | 93.09  ± 13.27 | 69.22  ± 23.58 | 149.59  ± 22.32* | 124.92  ± 16.04* | 101.68  ± 16.98 | 69.56  ± 19.34 |
| Tri-oxygenated:  C_23_H_44_O_3_, C_23_H_46_O_3_ | 16.47  ± 8.09 | 23.50  ± 5.46 | 28.13  ± 3.89 | 28.77  ± 4.77 | 16.11  ± 3.62 | 15.36  ± 2.22 | 34.70  ± 7.55 | 21.14  ± 9.91 | 43.23  ± 7.24* | 26.54  ± 29.66 | 29.66  ± 5.87 | 16.90  ± 6.34 |

^1^Values represent mean ± SEM; one way ANOVA with post-hoc Tukey’s HSD, *p<0.05 when compared to pooled average; DART –MS is not able to distinguish between isobaric molecules, hence values for each CHC species represent the sum intensity of all stereoisomers.

**^2^**The average signal intensity calculated from of all *oeno-Gal4* transgenic lines.

**^3^**The average signal intensity calculated from of all *dsx-Gal4* transgenic lines.
