# Supplementary material for: Steroid Hormone Signaling Is Essential for Pheromone Production and Oenocyte Survival
Source: PLoS Genet. 2016 Jun 22;12(6):e1006126. doi: 10.1371/journal.pgen.1006126 (PMC4917198; doi:10.1371/journal.pgen.1006126)
Supplement: S4 Table — (DOCX) [file pgen.1006126.s010.docx]

Supplemental Table 4. GCMS analysis of CHC extracts from female transgenic lines identified from the primary DART MS screen.

|  | **Signal intensity^3,4^** | | | | | | | | | | |
| --- | --- | --- | --- | --- | --- | --- | --- | --- | --- | --- | --- |
| CHC species^1,2^ | ***dsx/+*** | ***dsx>CG1444*** | ***oeno/+*** | ***oeno>CG7400*** | ***oeno>CG17562*** | ***dsx/+*** | ***dsx>CG2781*** | ***dsx>CG6300*** | ***oeno/+*** | ***oeno>CG9102*** | ***oeno>CG11502*** |
| C21:0 (nC21) | 0.19±0.002 | 0.66±0.04* | 0.33±0.01 | 0.11±0.002* | 0.19±0.02* | 0.19±0.01 | 0.11±0.002* | 0.09±0.01* | 0.30±0.02 | 0.15±0.004* | 0.15±0.02* |
| C22:1 | 0.003±0.003 | 0.05±0.05 | 0.02±0.0003 | 0.00* | 0.00* | 0.01±0.01 | 0 | 0 | 0.45±0.42 | 0 | 0 |
| 7,11-C23:2 | 0.21±0.21 | 0 | 0 | 0.06±0.05 | 0 | 0.55±0.06 | 0.13±0.002* | 0.71±0.05 | 0.74±0.13 | 0.22±0.02* | 1.11±0.33 |
| 9-C23:1 (9-T) | 0.21±0.003 | 0.32±0.01* | 0.30±0.01 | 0.05±0.001*** | 0.06±0.01*** | 0.30±0.02 | 0.09±0.004* | 0.02±0.02* | 0.27±0.04 | 0.08±0.003* | 0.15±0.01 |
| 7-C23:1 (7-T) | 1.84±0.06 | 0.62±0.02*** | 2.21±0.03 | 0.53±0.01*** | 0.42±0.14* | 2.23±0.05 | 1.01±0.04*** | 0.36±0.04*** | 1.86±0.23 | 0.98±0.04* | 1.58±0.05 |
| 5-C23:1 (5-T) | 0.19±0.01 | 0.07±0.03* | 0.44±0.004 | 0.04±0.01*** | 0.07±0.01*** | 0.22±0.03 | 0.08±0.01* | 0.00* | 3.76±3.38 | 0.21±0.01 | 0.32±0.002 |
| C23:0 (nC23) | 5.56±0.12 | 5.40±0.09 | 11.19±1.13 | 3.93±0.06* | 6.49±0.19* | 7.24±0.24 | 5.41±0.06* | 2.71±0.05*** | 10.84±0.78 | 7.94±0.06* | 5.87±0.68* |
| C24:2 | 0.09±0.01 | 0.00* | 0.19±0.01 | 0.01±0.01* | 0.08±0.03* | 0.07±0.02 | 0.07±0.01 | 0.00* | 0.16±0.02 | 0.05±0.01* | 0.17±0.01 |
| C24:1 | 0.11±0.0.1 | 0.00* | 0.10±0.01 | 0.01±0.01* | 0.01±0.01* | 0.13±0.02 | 0.05±0.001* | 0.03±0.03* | 0.22±0.13 | 0.05±0.001 | 0.07±0.01 |
| C24:0 | 0.52±0.01 | 0.46±0.02 | 1.36±0.15 | 0.35±0.001* | 1.02±0.01 | 0.51±0.01 | 0.47±0.01 | 0.45±0.01* | 1.13±0.12 | 0.61±0.003* | 1.03±0.02 |
| 7,11-C25:2 (7,11-PD);  2-MeC24 | 0.52±0.01 | 0.46±0.02* | 8.62±2.33 | 0.57±0.01* | 4.18±0.42 | 3.04±0.26 | 0.50±0.09* | 2.85±0.17 | 8.86±1.41 | 1.78±0.19* | 13.88±2.41 |
| 9-C25:1 (9-P) | 5.00±0.07 | 1.97±0.16*** | 7.07±0.09 | 2.47±0.02*** | 4.46±0.25* | 6.83±0.26 | 4.54±0.03* | 2.46±0.07*** | 7.46±0.48 | 5.55±0.18* | 8.62±0.13 |
| 7-C25:1 (7-P) | 6.22±0.01 | 1.53±0.20*** | 5.29±0.06 | 2.52±0.05*** | 2.40±0.05*** | 9.68±0.20 | 5.18±0.04*** | 2.38±0.18*** | 5.36±0.48 | 4.71±0.13 | 4.28±0.37 |
| 5-C25:1 (5-P) | 0.57±0.02 | 0.11±0.02*** | 0.64±0.03 | 0.16±0.02* | 0.35±0.02* | 0.71±0.03 | 0.76±0.01 | 0.06±.04* | 0.59±0.06 | 0.55±0.01 | 0.40±0.04 |
| C25:0 (nC25) | 5.22±0.08 | 6.07±0.07 | 13.43±1.77 | 5.99±0.09* | 10.65±0.08 | 7.62±0.40 | 8.83±0.17 | 6.13±0.13* | 13.40±0.78 | 11.58±0.01 | 11.15±1.35 |
| 7,11-C27:2 (7,11-HD);  2-MeC26 | 42.34±0.20 | 28.71±1.89* | 33.58±2.57 | 18.42±0.41 | 31.48±1.17* | 36.08±1.86 | 25.23±0.39* | 41.76±0.84* | 31.84±1.49 | 24.22±0.12* | 38.27±1.17* |
| 9-C27:1 | 3.65±0.04 | 5.50±0.38* | 1.60±0.36 | 6.61±0.1* | 3.60±0.37* | 4.64±0.38 | 5.46±0.08 | 6.76±0.12* | 2.13±0.10 | 3.70±0.13* | 1.49±0.40 |
| 7-C27:1 | 5.19±0.03 | 2.18±1.21 | 1.75±0.29 | 8.50±0.23*** | 3.79±0.45* | 7.68±1.05 | 11.49±0.22* | 7.79±0.40 | 1.99±0.15 | 4.53±0.20* | 1.62±0.47 |
| C27:0 (nC27) | 3.80±0.11 | 9.87±0.32*** | 3.70±0.75 | 14.02±0.26* | 6.20±0.11* | 4.45±0.28 | 10.84±0.15*** | 7.44±0.50* | 2.55±0.27 | 16.00±0.30*** | 3.28±0.59 |
| 7,11-C29:2 (7,11-ND)  2-MeC28 | 14.66±0.11 | 28.01±0.36*** | 7.08±0.58 | 26.82±0.18*** | 21.10±0.63*** | 7.35±0.45 | 17.78±0.45*** | 17.33±0.15*** | 4.52±0.35 | 11.99±0.15*** | 5.89±0.20* |
| C29:0 (nC29) | 0.28±0.003 | 2.05±0.19* | 0.30±0.06 | 4.71±0.15*** | 0.85±0.04* | 0.14±0.01 | 1.07±0.10* | 0.28±0.03* | 0.33±0.15 | 3.88±0.34* | 0.14±0.02 |
| 2-MeC30 | 0.41±0.02 | 3.19±0.13*** | 0.20±0.02 | 3.99±0.08*** | 2.22±0.21* | 0.07±0.01 | 0.55±0.05* | 0.20±0.06 | 0.52±0.23 | 0.98±0.05 | 0.26±0.02 |

^1^Elemental composition is represented by the carbon chain length followed by the number of double bonds.

^2^T: tricosene; P: pentacosene; PD: pentacosadiene; HD: heptacosadiene; ND: nonacosadiene; 2-Me: methyl branched alkanes.

^3^The signal intensity is calculated as the area of each peak divided by the total area of all hydrocarbons measured. Parallel sets of controls (*dsx/+* or *oeno*/+) were measured for each sample set. Values represent mean ± SEM, N=3; one-way ANOVA with post-hoc Tukey’s HSD, *p<0.05 when compared to *dsx*/+ or *oeno/+*; ***p<0.0001 when compared to controls.

^4^Signals corresponding to the long chain dienes and methyl branched CHCs could not be cleanly differentiated.
