# Supplementary material for: Steroid Hormone Signaling Is Essential for Pheromone Production and Oenocyte Survival
Source: PLoS Genet. 2016 Jun 22;12(6):e1006126. doi: 10.1371/journal.pgen.1006126 (PMC4917198; doi:10.1371/journal.pgen.1006126)
Supplement: S5 Table — (DOCX) [file pgen.1006126.s011.docx]

Supplemental Table 5. GCMS analysis of CHC extracts from male transgenic lines identified from the primary DART MS screen.

|  | **Signal intensity^3^** | | | | | | | | | | | | |
| --- | --- | --- | --- | --- | --- | --- | --- | --- | --- | --- | --- | --- | --- |
| CHC species^1,2^ | ***dsx/+*** | ***dsx>***  ***CG2781*** | ***dsx>***  ***CG7400*** | ***oeno/+*** | ***oeno>***  ***CG2781*** | ***oeno>***  ***CG7400*** | ***dsx/+*** | ***dsx>***  ***CG1765*** | ***dsx>***  ***CG11140*** | ***oeno/+*** | ***oeno>***  ***CG1444*** | ***oeno>***  ***CG5162*** | ***oeno>***  ***CG8522*** |
| C21:0 (nC21) | 0.58±0.01 | 0.64±0.05 | 0.46±0.02* | 1.27±0.01 | 0.81±0.02*** | 0.40±0.02*** | 0.55±0.01 | 0.42±0.02* | 1.35±0.04*** | 1.09±0.07 | 1.34±0.13 | 1.38±0.06* | 0.31±0.02* |
| C22:1 | 0.29±0.01 | 0.34±0.01* | 0.24±0.01* | 0.44±0.01 | 0.49±0.02* | 0.23±0.03* | 0.31±0.01 | 0.27±0.01* | 0.33±0.01 | 0.47±0.004 | 0.30±0.02* | 0.61±0.01*** | 0.26±0.03* |
| cVA | 0.48±0.14 | 0.92±0.28 | 0.53±0.07 | 0.19±0.03 | 0.69±0.08* | 0.38±0.01* | 0.74±0.11 | 1.49±0.22* | 0.52±0.09 | 0.76±0.12 | 1.72±0.30* | 1.53±0.31 | 1.72±0.78 |
| 7,11-C23:2 | 0.01±0.01 | 0 | 0 | 0.02±0.002 | 0.02±0.01 | 0.00* | 0.10±0.002 | 0.05±0.002* | 0.09±0.01 | 0.26±0.03 | 1.43±0.45 | 0.30±0.02 | 0.12±0.02* |
| 9-C23:1 (9-T) | 2.19±0.01 | 1.84±0.10* | 1.57±0.05* | 2.54±0.03 | 3.38±0.07* | 1.10±0.06*** | 2.75±0.07 | 2.22±0.15* | 2.77±0.05 | 2.67±0.16 | 1.67±0.30* | 2.40±0.12 | 1.21±0.06* |
| 7-C23:1 (7-T) | 27.68±0.77 | 26.48±0.20 | 20.72±0.57* | 36.08±0.27 | 30.59±0.83* | 14.60±0.61*** | 29.95±0.08 | 25.75±0.88* | 36.16±0.53* | 35.47±0.78 | 20.40±1.17* | 33.10±0.77 | 22.72±2.25* |
| 5-C23:1 (5-T) | 3.09±0.11 | 2.41±0.01* | 1.95±0.05* | 3.19±0.01 | 2.57±0.05* | 1.83±0.08*** | 2.80±0.06 | 2.35±0.10* | 3.36±0.04* | 3.15±0.11 | 1.80±0.10* | 3.32±0.05 | 3.06±0.09 |
| C23:0 (nC23) | 11.45±0.45 | 13.25±0.94 | 11.04±0.11 | 18.47±0.44 | 13.19±0.48* | 16.68±0.42* | 12.21±0.22 | 10.57±0.07* | 13.93±0.19* | 18.95±0.81 | 13.78±0.24* | 19.76±0.17 | 15.61±0.34* |
| C24:2 | 0.68±0.03 | 0.73±0.03 | 0.59±0.02 | 1.16±0.06 | 1.34±0.01* | 0.58±0.09* | 0.66±0.03 | 0.62±0.03 | 0.49±0.01* | 0.86±0.07 | 0.57±0.07* | 1.38±0.03* | 1.02±0.04 |
| C24:1 | 0.97±0.03 | 0.89±0.02 | 0.82±0.02* | 0.63±0.01 | 0.64±0.003 | 0.56±0.03 | 0.98±0.03 | 1.04±0.04 | 0.72±0.01* | 0.67±0.03 | 0.44±0.05* | 0.70±0.10 | 1.03±0.07* |
| C24:0 | 0.29±0.004 | 0.48±0.01* | 0.41±0.01* | 0.72±0.003 | 0.55±0.02* | 0.96±0.04* | 0.34±0.01 | 0.38±0.02 | 0.28±0.003* | 0.60±0.04 | 0.65±0.02 | 0.91±0.02* | 1.01±0.06* |
| 2-MeC24 | 0.77±0.02 | 093±0.16 | 0.69±0.01* | 2.57±0.06 | 3.04±0.06* | 2.39±0.05 | 0.77±0.01 | 0.79±0.04 | 0.37±0.03* | 3.71±0.10 | 5.83±1.02 | 3.50±0.19 | 3.03±0.25 |
| 9-C25:1 (9-P) | 5.90±0.25 | 4.75±0.31* | 5.15±0.06* | 3.57±0.12 | 6.36±0.19* | 6.85±0.18* | 5.76±0.08 | 5.77±0.25 | 3.39±0.04*** | 4.51±0.19 | 2.85±0.30* | 4.34±0.18 | 6.56±0.33* |
| 7-C25:1 (7-P) | 31.17±0.48 | 24.62±0.52* | 28.80±0.38* | 12.10±0.27 | 11.74±0.59 | 21.03±0.37*** | 28.14±0.53 | 29.00±0.52 | 23.42±0.67* | 13.02±0.11 | 9.49±0.93* | 12.20±1.01 | 23.18±1.67* |
| 5-C25:1 (5-P) | 0.98±0.05 | 0.55±0.03* | 0.71±0.02* | 0.28±0.02 | 0.24±0.01 | 0.81±0.01*** | 0.70±0.02 | 0.65±0.002* | 0.17±0.01*** | 0.18±0.07 | 0.28±0.02 | 0.23±0.04 | 0.84±0.12* |
| C25:0 (nC25) | 2.26±0.02 | 2.80±0.09* | 3.24±0.07* | 3.10±0.05 | 2.58±0.03* | 6.06±0.28* | 2.77±0.10 | 3.27±0.02* | 2.60±0.03 | 3.34±0.21 | 2.97±0.13 | 3.35±0.07 | 4.88±0.31* |
| 2-MeC26 | 1.89±0.03 | 3.83±1.32 | 3.55±0.08*** | 5.49±0.23 | 8.49±0.29* | 7.09±0.31* | 3.89±0.02 | 5.07±0.17* | 2.09±0.10*** | 5.85±0.63 | 16.46±1.59* | 5.33±0.16 | 6.06±0.29 |
| 9-C27:1 | 0.35±0.03 | 0.29±0.01 | 0.61±0.01* | 0.05±0.03 | 0.09±0.02 | 0.31±0.02* | 0.25±0.03 | 0.46±0.03* | 0.14±0.01* | 0.09±0.01 | 0.11±0.06 | 0.01±0.01* | 0.09±0.04 |
| 7-C27:1 | 1.59±0.11 | 1.09±0.18 | 2.30±0.08* | 0.17±0.01 | 0.20±0.01 | 1.32±0.05*** | 0.77±0.02 | 1.28±0.08* | 0.50±0.03* | 0.09±0.01 | 0.23±0.06 | 0.13±0.02 | 0.33±0.07* |
| C27:0 (nC27) | 2.58±0.06 | 3.65±0.44 | 5.06±0.15* | 1.43±0.04 | 1.38±0.06 | 4.55±0.18*** | 2.06±0.07 | 3.00±0.13* | 2.39±0.06* | 0.74±0.07 | 1.41±0.17* | 1.09±0.00* | 2.01±0.13* |
| 2-MeC28 | 2.43±0.06 | 5.84±1.33 | 6.75±0.15*** | 4.08±0.20 | 8.27±0.23* | 7.99±0.57* | 2.33±0.05 | 4.17±0.59* | 3.14±0.13* | 1.78±0.33 | 11.67±0.09*** | 1.83±0.09 | 3.42±0.30* |
| C29:0 (nC29) | 1.11±0.06 | 1.69±0.48 | 2.42±0.07* | 0.22±0.01 | 0.30±0.04 | 1.35±0.11* | 0.26±0.02 | 0.48±0.08 | 0.59±0.02* | 0.03±0.01 | 0.21±0.06* | 0.09±0.00* | 0.22±0.03* |
| 2-MeC30 | 0.49±0.03 | 1.01±0.02* | 1.61±0.08* | 0.27±0.02 | 1.19±0.16* | 1.85±0.17* | 0.11±0.004 | 0.20±0.07 | 0.28±0.01* | 0.03±0.02 | 1.97±0.28* | 0.06±0.03 | 0.13±0.01* |

^1^Elemental composition is represented by the carbon chain length followed by the number of double bonds.

^2^T: tricosene; P: pentacoseane; 2-Me: methyl branched alkanes.

^3^The signal intensity is calculated as the area of each peak divided by the total area of all hydrocarbons measured. Parallel sets of controls (*dsx/+* or *oeno*/+) were measured for each sample set. Values represent mean ± SEM, N=3; one-way ANOVA with post-hoc Tukey’s HSD, *p<0.05 when compared to *dsx*/+ or *oeno/+*; ***p<0.0001 when compared to controls.
