# Supplementary material for: Steroid Hormone Signaling Is Essential for Pheromone Production and Oenocyte Survival
Source: PLoS Genet. 2016 Jun 22;12(6):e1006126. doi: 10.1371/journal.pgen.1006126 (PMC4917198; doi:10.1371/journal.pgen.1006126)
Supplement: S6 Table — (DOCX) [file pgen.1006126.s012.docx]

**Supplemental Table 6.** Genotypes used for each figure.

| **Figure** | **Genotype** | **Drug regimen** |
| --- | --- | --- |
| Figure 2 | *w; +; oeno-Gal4, tubGal80^ts^*  *w; UAS-CG1444-RNAi^(1)^; oeno-Gal4, tubGal80^ts^*  *w; +; oeno-Gal4, tubGal80^ts^, UAS-CG1444-3XHA*  *w; +; oeno-Gal4, tubGal80^ts^, UAS-ORF-CG1444* |  |
| Figure 3 | *w; GSoeno-Gal4, UAS-CG1444-RNAi^(1)^*  *w; +; oeno-Gal4, tubGal80^ts^*  *w; +; oeno-Gal4, tubGal80^ts^, UAS-CG1444-3XHA* | *spidey^control^*: - RU486  *spidey^KD^*: + RU486 |
| Figure 4A-D | *w; GSoeno-Gal4, UAS-CG1444-RNAi^(1)^* | *spidey^control^*: - RU486  *spidey^KD^*: + RU486 |
| Figure 4E, F | *w; GSoeno-Gal4, UAS-CG1444-RNAi ^(1)^*  *w; +; oeno-Gal4, tubGal80^ts^*  *w; UAS-CG1444-RNAi^(1)^; oeno-Gal4, tubGal80^ts^*  *w; UAS-CG1444-RNAi^(2)^; oeno-Gal4, tubGal80^ts^*  *w; UAS-hid, stinger; oeno-Gal4, tubGal80^ts^*  *Wildtype (Canton-S)* | *spidey^control^*: - RU486  *spidey^KD^*: + RU486 |
| Figure 5 | *w; GSoeno-Gal4, UAS-CG1444-RNAi^(1)^* | *spidey^control^*: - RU486  *spidey^KD^*: + RU486 |
| Figure 6A, C | *w; UAS-CG1444-RNAi^(1)^; oeno-Gal4, tubGal80^ts^, UAS-mcd8:GFP*  *w; oeno-Gal4, tubGal80^ts^, UAS-mcd8:GFP* |  |
| Figure 6B, D | *w; UAS-CG1444-RNAi^(1)^; oeno-Gal4, tubGal80^ts^*  *w; oeno-Gal4, tubGal80^ts^* |  |
| Figure 7A | *w; UAS-CG1444-RNAi^(1)^; oeno-Gal4, tubGal80^ts^* |  |
| Figure 7B, C | *w; UAS-CG1444-RNAi^(1)^; oeno-Gal4, tubGal80^ts^, UAS-mcd8:GFP* |  |
| Supp. Figure 1 | *w; +; oeno-Gal4, tubGal80^ts^, UAS-mcd8:GFP*  *w; dsx-Gal4, UAS-mcd8:GFP* |  |
| Supp. Figure 2 | *w; +; oeno-Gal4, tubGal80^ts^, UAS-desat1-RNAi*  *w; +; oeno-Gal4, tubGal80^ts^, UAS-desatF-RNAi*  *w; +; oeno-Gal4, tubGal80^ts^, UAS-eloF-RNAi*  *w; +; oeno-Gal4, tubGal80^ts^* |  |
| Supp. Figure 3 | *w; UAS-CG1444-RNAi^(1)^; oeno-Gal4, tubGal80^ts^* |  |
| Supp. Figure 4 | *Wildtype (Canton-S)* |  |
| Supp. Figure 5A,B | *w; GSoeno-Gal4, UAS-CG1444-RNAi^(1)^* | *spidey^control^*: - RU486  *spidey^KD^*: + RU486 |
| Supp. Figure 5C, D | *w; GStubulin-Gal4, UAS-CG1444-RNAi^(1)^* | *spidey^tubcontrol^*: - RU486  *spidey^tubKD^*: + RU486 |
